# Bottom-Up Synthesis of SnTe-Based Thermoelectric Composites

**DOI:** 10.1021/acsami.3c00625

**Published:** 2023-05-04

**Authors:** Bingfei Nan, Xuan Song, Cheng Chang, Ke Xiao, Yu Zhang, Linlin Yang, Sharona Horta, Junshan Li, Khak Ho Lim, Maria Ibáñez, Andreu Cabot

**Affiliations:** † Catalonia Institute for Energy Research—IREC, Sant Adrià de Besòs, Barcelona 08930, Spain; ‡ Universitat de Barcelona, Martí i Franquès 1, Barcelona 08028, Spain; § The State Key Laboratory of Chemical Engineering, Department of Chemical Engineering, Tsinghua University, Beijing 100084, China; ∥ Institute of Science and Technology Austria (ISTA), Am Campus 1, Klosterneuburg 3400, Austria; ⊥ School of Materials Science and Engineering, Beihang University, Beijing 100191, China; # Department of Materials Science and Engineering, Pennsylvania State University, University Park, State College, Pennsylvania 16802, United States; ¶ Institute of Advanced Study, Chengdu University, Chengdu 610106, China; ∇ Institute of Zhejiang University—Quzhou, 99 Zheda Rd, Quzhou 324000, Zhejiang, P. R. China; ○ College of Chemical and Biological Engineering, Zhejiang University, 38 Zheda Rd, Hangzhou 310007, Zhejiang, P. R. China; ⧫ ICREA, Pg. Lluís Companys 23, Barcelona 08010, Catalonia, Spain

**Keywords:** tin telluride, copper telluride, thermoelectric, Cu_2_SnTe_3_, molecular precursor, nanocomposite

## Abstract

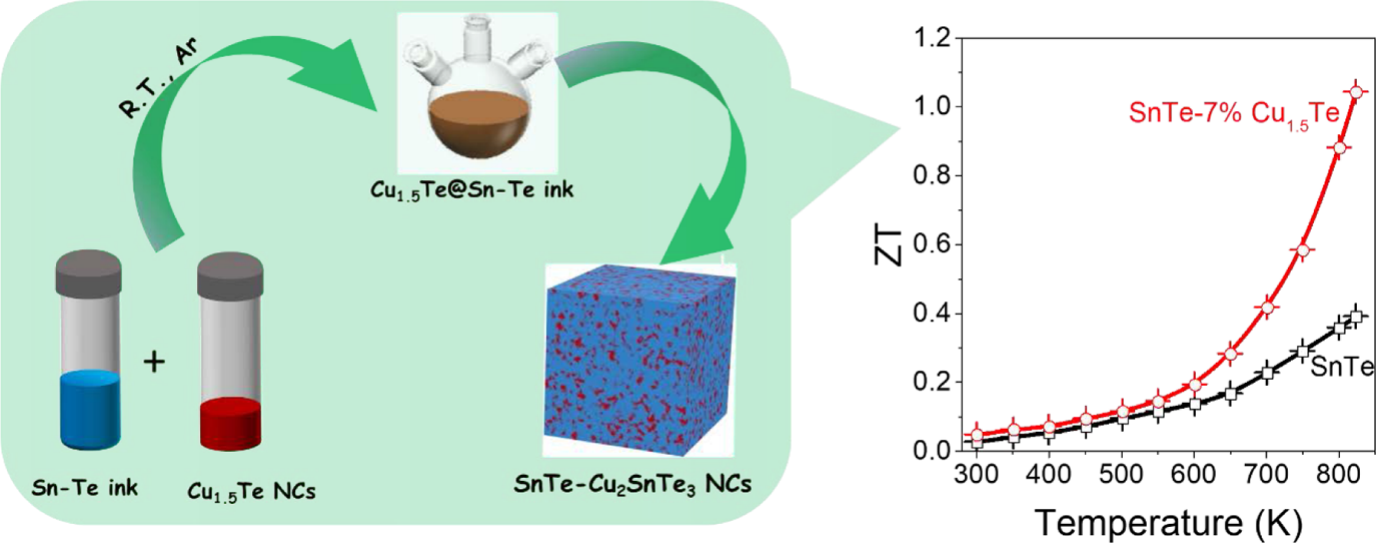

There is a need for the development of lead-free thermoelectric materials for medium-/high-temperature applications. Here, we report a thiol-free tin telluride (SnTe) precursor that can be thermally decomposed to produce SnTe crystals with sizes ranging from tens to several hundreds of nanometers. We further engineer SnTe–Cu_2_SnTe_3_ nanocomposites with a homogeneous phase distribution by decomposing the liquid SnTe precursor containing a dispersion of Cu_1.5_Te colloidal nanoparticles. The presence of Cu within the SnTe and the segregated semimetallic Cu_2_SnTe_3_ phase effectively improves the electrical conductivity of SnTe while simultaneously reducing the lattice thermal conductivity without compromising the Seebeck coefficient. Overall, power factors up to 3.63 mW m^–1^ K^–2^ and thermoelectric figures of merit up to 1.04 are obtained at 823 K, which represent a 167% enhancement compared with pristine SnTe.

## Introduction

Thermoelectric (TE) materials enable the direct and reversible conversion between thermal and electrical energies.^1^ Owing to the omnipresence of thermal energy, the development of TE devices to power wireless sensors, recover waste heat, and regulate temperature is attracting increasing attention.^2,3^ Toward harvesting energy, while thermal energy is ubiquitous, the energy conversion efficiency of the TE device determines its size and thus cost-effectiveness. Aside from thermal and electric contacts, this energy conversion efficiency can be estimated from a dimensionless figure of merit (ZT) of the TE material, defined as , where *S*, σ, *T*, and κ_tot_ are, respectively, the Seebeck coefficient, electrical conductivity, absolute temperature, and total thermal conductivity that includes a lattice κ_L_ and an electronic κ_e_ component. Unfortunately, the interdependencies among these parameters make the optimization of TE materials an extremely difficult task.

TE parameters strongly depend on the temperature. While ambient temperature applications are dominated by devices based on Bi–Sb–Te–Se alloys,^4−7^ in the medium-/high-temperature range (600–800 K), lead chalcogenides provide the highest ZT values.^8−11^ However, the presence of toxic Pb in these materials is a major drawback toward commercialization.^12,13^ Tin telluride (SnTe), a group IV–VI semiconductor with similar crystal and band structures to PbTe, is a particularly suitable alternative TE material for the medium-/high-temperature range.^14−16^ Nevertheless, a too large carrier concentration (∼10^20^ to 10^21^ cm^–3^) originating from a high density of Sn vacancies, a too-narrow band gap (∼0.18 eV at 300 K), and a high energy offset (∼0.35 eV at 300 K) between the light valence band L and the heavy valence band Σ result in elevated thermal conductivities and moderate Seebeck coefficients and thus an overall poor TE performance.^17,18^

Several strategies have been developed to improve the TE properties of SnTe, including energy filtering,^19,20^ band convergence,^21,22^ hyperconvergence,^23,24^ Rashba effect,^25^ introduction of resonant levels,^12^ modulation of defects,^26^ incorporation of dense dislocations and precipitates,^27^ and combinations of these approaches.^13,28−31^ Within these strategies, the incorporation of dopants and alloying phases into the SnTe matrix to form point defects and/or precipitates that act as strong phonon scattering centers is particularly effective. These phases include In_2_Te_3_,^32^ CdSe,^33^ Cu_1.75_Se,^34^ Cu_2_Te,^35^ SnS,^36^ AgBiTe_2_,^37^ MgAgSb,^38^ and CuSbSe_2_.^39^ In particular, copper has been introduced in an ionic form within the SnTe matrix and as segregated Cu-based phases. Both interstitial Cu atoms and segregated Cu_2_Te/Cu_1.75_Te phases have been shown to effectively scatter phonons, resulting in extremely low lattice thermal conductivities down to 0.5 W m^–1^ K^–1^.^40−44^

Synthetic approaches developed for SnTe-based materials include the high-temperature melting method,^13,45,46^ mechanical alloying,^47^ melt spinning,^48^ self-propagating high-temperature synthesis,^49,50^ solvothermal method,^51−54^ microwave method,^55,56^ and aqueous solution method.^57^ Among them, the bottom-up engineering of composites using solution-processed nanocrystals as building blocks is a particularly scalable, low-cost, and extremely versatile approach to optimize the performance in numerous applications.^58−63^ Within this approach, the use of inorganic ligands or molecular precursors to adjust the nanocrystal surface composition and to produce nanocomposites with controlled phase distribution has emerged as an especially suitable strategy.^64−69^ In this direction, we have recently reported the preparation of tin chalcogenides such as SnSe_2_,^70^ SnSe,^71^ and SnS_2_^72^ using molecular precursor inks. Besides, we recently demonstrated that a CdSe ligand could act as a secondary phase during the nanocomposite consolidation to modify the electronic band structure of the SnTe matrix, reaching ZT values up to 1.3 at 850 K.^73^ We also recently demonstrated the effectiveness of a soluble PbS molecular complex in a thiol-amine solvent to modify the SnTe nanocrystal surface and produce SnTe–PbS nanocomposites, reaching ca. 0.8 ZT value at 873 K.^74^

Brutchey et al. showed a Sn–Te precursor solution dissolved using ethylenediamine and a thiol at a 4:1 volume rate, yielding crystalline SnTe with Te impurity after solution deposition and heat treatment at 250 °C.^75^ While thiols offer evident safety advantages over extensively used hydrazine, they should also be prevented owing to health and safety issues and the potential contamination of the final material with sulfur.

Herein, we demonstrate the preparation of a SnTe precursor using a thiol-free solvent based on oleylamine (OAm) and tri-*n*-octylphosphine (TOP). This precursor allows producing pure SnTe at moderate decomposition temperatures. In contrast to previously reported procedures to produce SnTe nanocrystals,^73,76,77^ the use of a Sn–Te precursor ink offers advantages in terms of the processability of printed SnTe-based devices. Besides, the SnTe ink can be combined with Cu_1.5_Te nanoparticles to produce Sn–Cu–Te nanocomposites upon thermal decomposition. Dense SnTe–Cu_2_SnTe_3_ nanocomposites are then obtained by a hot-press sintering process. Interestingly, the produced Cu_2_SnTe_3_ remarkably improves the power factor by increasing the electrical conductivity without deteriorating the Seebeck coefficient. At the same time, the lattice thermal conductivity is strongly decreased. As a result, record ZT values for this system are demonstrated.

## Results and Discussion

**Figure 1:**
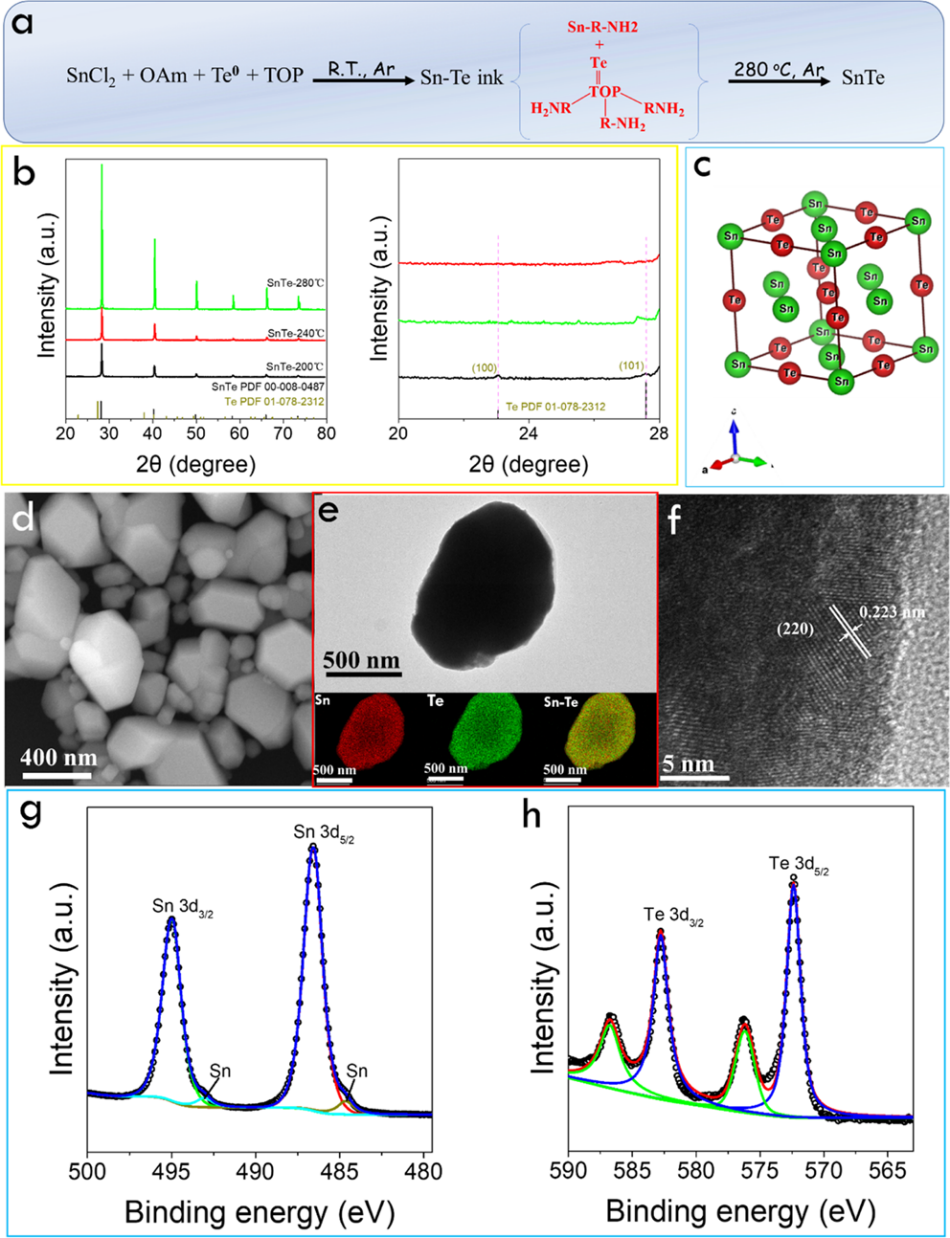
(a) Scheme of the formation of SnTe from the ink containing Sn–OAm and TOPTe complexes. (b) Powder XRD patterns of the material obtained from the SnTe precursor decomposed at different temperatures of 200, 240, and 280 °C. The graph on the right displays an expanded XRD pattern to show the presence of the Te phase. (c) Cubic rock-salt SnTe crystal phase. (d) SEM image of SnTe obtained at 280 °C. (e) TEM and EDX mapping of a SnTe particle. (f) HRTEM image of a SnTe particle. (g) and (h) High-resolution Sn 3d and Te 3d XPS spectra of SnTe obtained at 280 °C.

A SnTe precursor was prepared by coordinating Sn^2+^ ions with OAm to form a Sn–OAm complex and combining it with TOP telluride (TOPTe) (see details in the Supporting Information, Figures 1a and S1).^78,79^Figure 1b displays the X-ray diffraction (XRD) pattern of the materials obtained from the decomposition of the Sn–Te ink at different temperatures. The XRD patterns display the diffraction peaks corresponding to the (200), (220), (222), (400), (420), and (422) planes of the cubic rock-salt SnTe crystal belonging to the *Fm*3̅*m* space group with *a* = 6.303 Å (Figure 1c). Additional peaks corresponding to Te impurities are found in the material obtained at 200 °C. Upon increasing the decomposition temperature from 200 to 280 °C, the impurity peaks disappear and the intensity of the cubic SnTe pattern increases, indicating a more effective reaction of Te and an improved SnTe crystallization and/or larger SnTe crystal domains.

Scanning electron microscopy (SEM) characterization showed a notable increase in the particle size with the reaction temperature (Figure S2). As shown in Figures 1d and S3, the SnTe particles obtained at 280 °C displayed a highly faceted morphology and a broad size distribution with an average size of 300 ± 200 nm. Energy-dispersive X-ray (EDX) analysis results revealed a slight excess of Sn, Sn/Te ∼1.1 (Table S1). This uncommon Sn-rich composition has been previously obtained using colloidal synthesis strategies, probably owing to the stabilization of Sn-terminated surfaces.^80^ Previously synthesized pure SnTe particles usually exhibit octahedral-shaped structures with eight (111) planes because the surface energy of the (111) planes is lower than that of the (110) planes in Sn-poor SnTe particles.^81−83^ Unlike the (111) dominance plane from Sn-poor SnTe particles, the preferential planes in Te-rich particles should be the (110) planes.^84,85^ Transmission electron microscopy (TEM)-EDX maps showed a uniform distribution of Sn and Te at the nanometer scale (Figure 1e). Besides, high-resolution TEM (HRTEM) confirmed the cubic SnTe phase and the highly crystalline structure of the particles (Figure 1f).

Figure 1g,h displays the X-ray photoelectron spectroscopy (XPS) spectra of SnTe obtained at 280 °C. The high-resolution Sn 3d XPS spectrum exhibits one doublet at 495.1 eV (Sn 3d_3/2_) and 486.6 eV (Sn 3d_5/2_) associated with a Sn^2+^ oxidation state within a chalcogenide chemical environment.^45^ A second small doublet appears at 493.1 and 484.7 eV, and it is ascribed to the presence of a small amount of metallic Sn.^86^ The high-resolution Te 3d XPS spectrum exhibits two doublets. The main one is located at 582.8 eV (Te 3d_3/2_) and 572.4 eV (Te 3d_5/2_), and it is associated with Te^2–^ within a metal telluride chemical environment.^26^ The second one is located at significantly higher binding energies, 586.7 (Te 3d_3/2_) and 576.3 (Te 3d_5/2_), and it is associated with an oxidized component formed during the material’s exposure to ambient conditions for manipulation and transportation.

**Figure 2:**
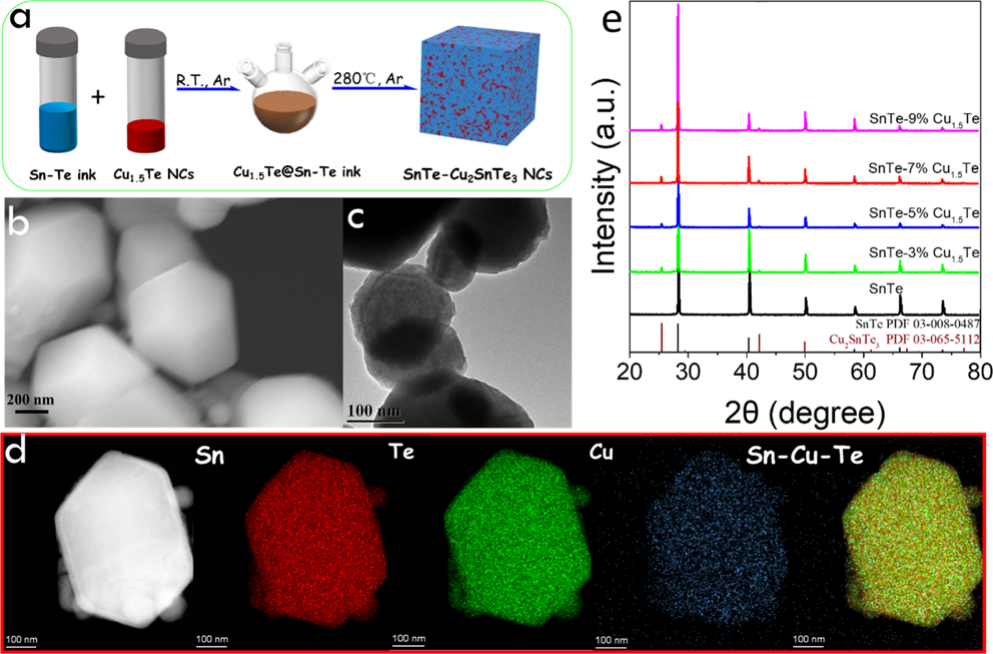
(a) Schematic diagram of the engineering of SnTe–Cu_2_SnTe_3_ composites from a combination of the Sn–Te ink (blue) and a colloidal dispersion of Cu_1.5_Te nanoparticles (red). (b) SEM and (c) TEM image of SnTe–7% Cu_1.5_Te composite, (d) HAADF image and EDX elemental maps of Sn, Te, and Cu. (e) XRD patterns of the different composites.

To produce SnTe-based composites, the SnTe molecular precursor was combined with different amounts of Cu_1.5_Te nanocrystals dispersed in OAm (Figures 2a and S1, see the Supporting Information for details on the synthesis of Cu_1.5_Te). The obtained solution was decomposed at 280 °C. All the synthesized SnTe-*y*% Cu_1.5_Te composite powders displayed similar particle morphologies as the pristine SnTe particles. Figure 2b,c displays representative SEM and TEM images of the SnTe–7% Cu_1.5_Te composite. EDX mapping shows a uniform distribution of Cu, Sn, and Te, denoting the presence of some amount of Cu within the SnTe particles (Figure 2d). Figure 2e shows the XRD pattern of the different samples. The main diffraction peaks can be indexed as the rock-salt structure of SnTe (PDF 00-008-0487). Besides, additional XRD peaks are visible at 25.5 and 42.2° and correspond to the (111) and (220) family planes of the Cu_2_SnTe_3_ cubic phase (PDF 03-065-5112). No Cu_2–*x*_Te phase could be detected by XRD. The disappearance of the Cu_1.5_Te phase, the ubiquitous presence of Cu, and the appearance of a small amount of Cu_2_SnTe_3_ indicate the Cu diffusion within SnTe particles and the segregation of a ternary Cu–Sn–Te phase from the partial reaction of Sn and Te precursors with Cu_1.5_Te. The SnTe XRD peak positions within the SnTe-*y*% Cu_1.5_Te composite are influenced by the presence of Cu, further evidencing the presence of Cu within the SnTe lattice. As observed in Figure S4, with the increase of the Cu_1.5_Te amount within the initial precursor, the (200) XRD peak initially shifts to notably lower angles, probably due to the presence of interstitial Cu within SnTe, and then slightly returns toward its initial position because of the segregation of the Cu_2_SnTe_3_ phase, decreasing the amount of Cu within the SnTe lattice.

**Figure 3:**
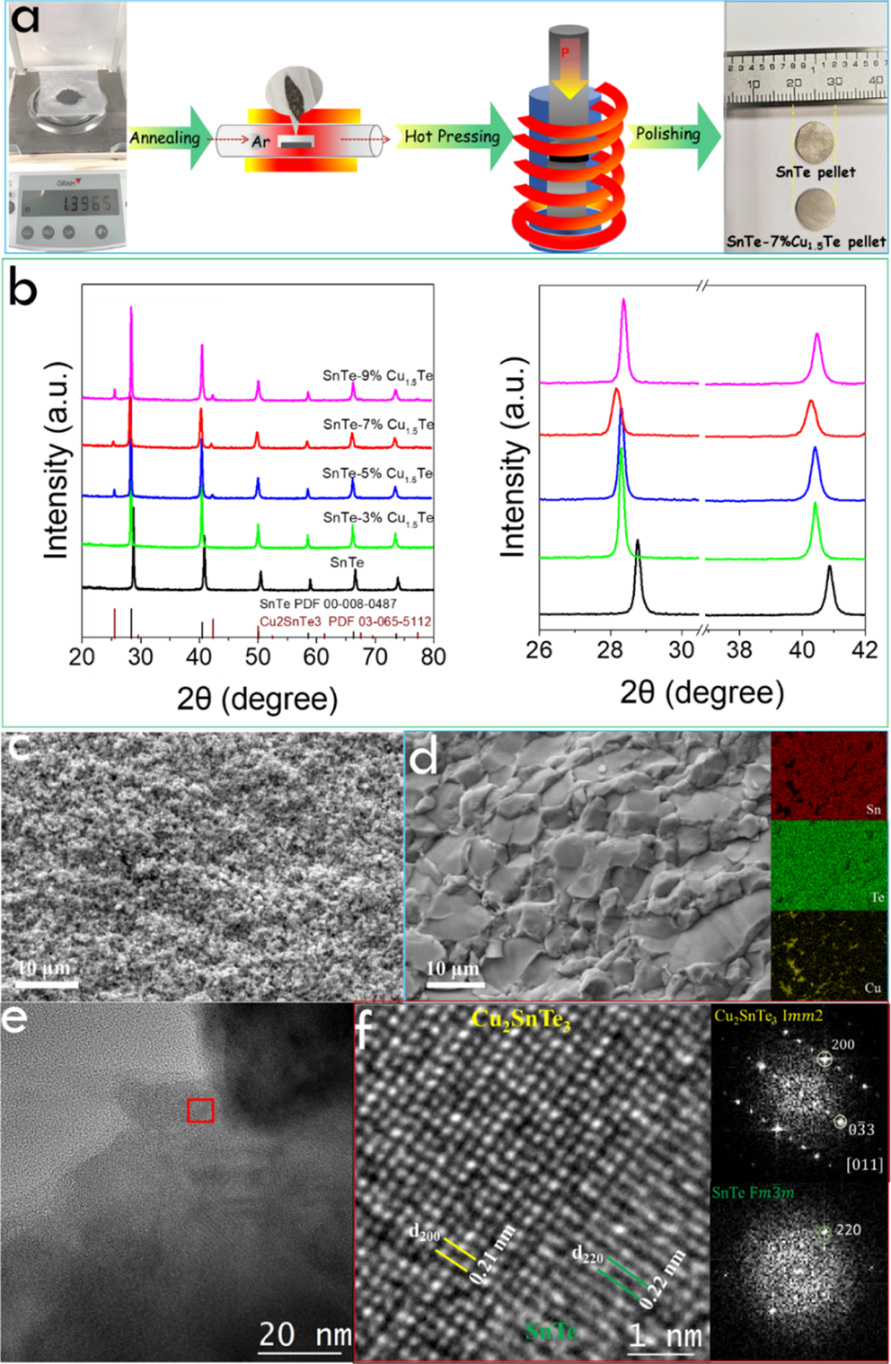
(a) Schematic illustration of the hot pressing of the annealed composites into a pellet. (b) XRD patterns of sintered SnTe-*y*% Cu_1.5_Te pellets (*y* = 0, 3, 5, 7, and 9%) and enlarged (200) and (220) peaks. (c) Cross-sectional SEM image of the sintered SnTe pellet. (d) Cross-section SEM image of the sintered SnTe–7%Cu_1.5_Te pellet and corresponding EDX maps of Sn, Te, and Cu. (e) HRTEM image of SnTe–7% Cu_1.5_Te pellet. (f) HRTEM image of the red area in (e), and FFT from SnTe and Cu_2_SnTe_3_ different phase regions, respectively.

As schematized in Figure 3a, to evaluate the TE properties of SnTe and SnTe-*y*% Cu_1.5_Te, the annealed powders (853 K for 120 min under Ar, see details in the Supporting Information) were sintered into disk-shaped pellets by hot pressing under 40 M uniaxial pressure at 773 K for 5 min in an argon-filled glovebox (see details in the Supporting Information). The relative densities of the sintered pellets determined by the Archimedes method were all above 97% (Table S2, Supporting Information). Figure 3b shows the XRD patterns of sintered SnTe and SnTe-*y*% Cu_1.5_Te (*y* = 3, 5, 7, and 9%) pellets. The Cu_2_SnTe_3_ secondary phase can be detected only when the precursor Cu_1.5_Te concentration is ≥5%. As for the sintered powder, when increasing the precursor Cu_1.5_Te concentration, the SnTe XRD patterns show first a strong shift to lower diffraction angles that it is partially recovered at higher Cu_1.5_Te concentrations. We assign the XRD peak shift toward a higher angle position with the precursor Cu_1.5_Te content from 7 to 9% with the segregation of the Cu_2_SnTe_3_ phase, reducing the amount of Cu within the SnTe lattice.

To visualize the internal microstructure and morphology of the sintered samples, representative SEM images of the fractured pellets are displayed in Figures 3c,d and S5–S7. The cross-sectional SEM micrographs of SnTe exhibit small grains. Compared with pristine SnTe, the presence of Cu_1.5_Te in the thermally decomposed precursor solution boosts the crystal growth during the thermal processes, increasing the crystal domain size of the final SnTe-*y*% Cu_1.5_Te composites by more than one order of magnitude. The large grain growth is mainly assigned to the hot-pressing sintering process instead of the annealing process (Figure S6c,e). The enhanced crystal growth is related to the low melting point of the Cu_2_SnTe_3_ phase formed at 680 K.^87^ As the melting point of Cu_2_SnTe_3_ is below that used during hot pressing, the Cu_2_SnTe_3_ phase melts during the process and acts as a solvent, promoting the diffusion of Sn and Te atoms and promoting the SnTe grain growth to micron-sized grains.^74^ No preferential growth or orientation of the crystals within the pellets was observed either by SEM analysis or XRD analysis (Figure S7), which points toward a material having anisotropic properties.

EDX elemental maps show a homogeneous distribution of Sn, Te, and Cu throughout the whole pellet at a low (3%) Cu_1.5_Te concentration. In contrast, Cu-rich areas are found in the composites obtained from larger amounts of precursor Cu_1.5_Te, 5, 7, and 9%. This observation is consistent with the XRD results. Taking into account the XRD data, we associate the local Cu accumulation in the SnTe matrix with the presence of nanoprecipitates of the Cu_2_SnTe_3_ phase. The presence of Cu_2_SnTe_3_ crystal domains within the composites was further confirmed by HRTEM. As shown in Figure 3e,f, a d-spacing of 0.22 nm is assigned to the (220) planes of the SnTe matrix. Besides, Cu_2_SnTe_3_ nanodomains are identified with a d-spacing of 0.21 nm, which corresponds to the (200) plane of the Cu_2_SnTe_3_ phase. The size of the Cu_2_SnTe_3_ nanodomains observed on the HRTEM image is ca. 10 nm (Figure S8a,b). HAADF-EDX maps show a relatively uniform distribution of Sn and Te elements, while some Cu-rich areas are evident (Figure S8c).

**Figure 4:**
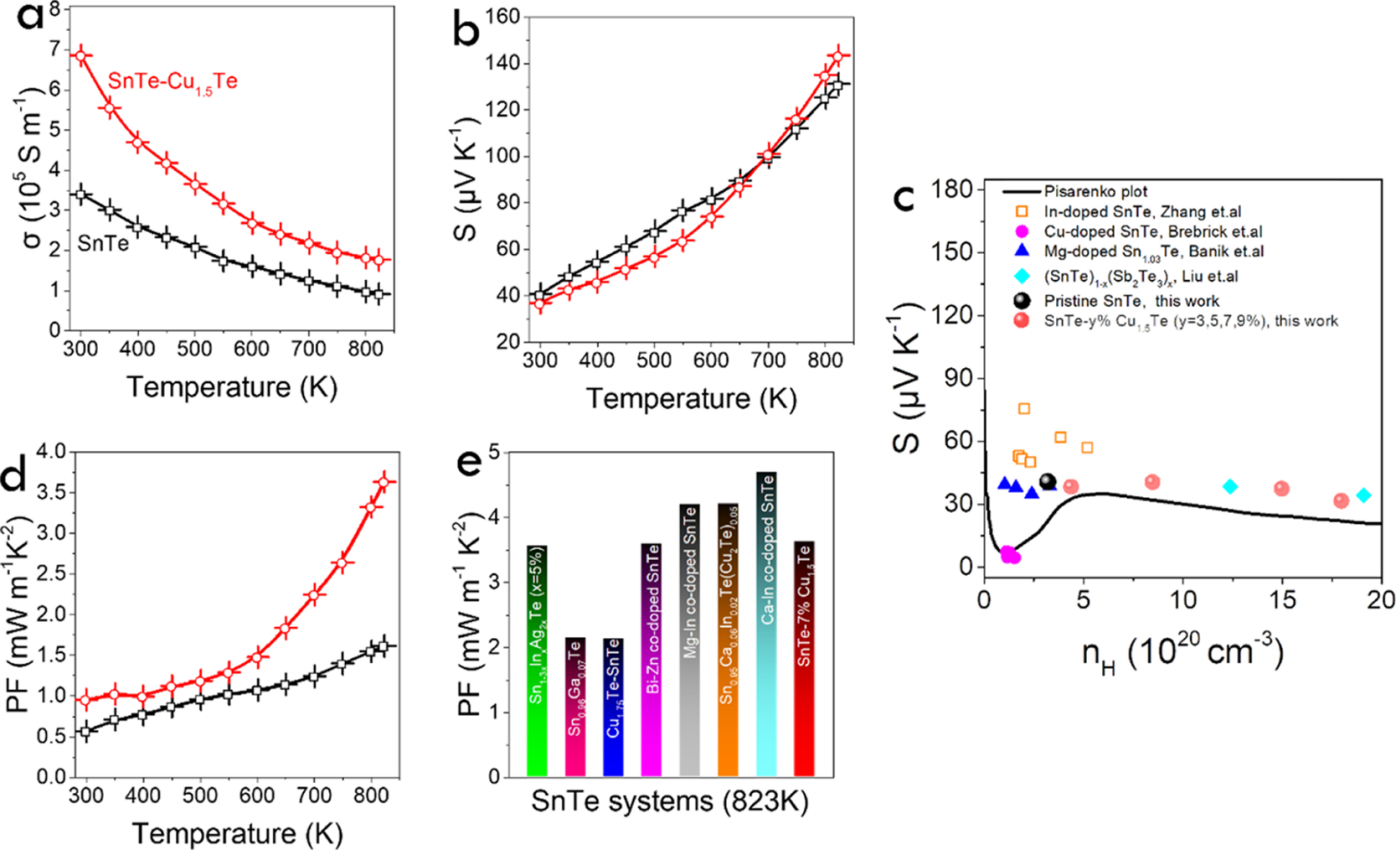
(a,b) Temperature dependence of (a) electrical conductivity, σ. (b) Seebeck coefficient, *S*. (c) Room temperature Seebeck coefficient (*S*) as a function of Hall carrier concentrations (*n*_H_). The solid line is the room temperature theoretical Pisarenko plot calculated using a two-valence-band model. Previously reported In-doped SnTe,^12^ Cu-doped SnTe,^92^ Mg-doped Sn_1.03_Te,^93^ and (SnTe)_1–*x*_(Sb_2_Te_3_)_*x*_^94^ are listed for comparison. (d) Power factor, *S*^2^σ, PF for the pristine SnTe and SnTe-7% Cu_1.5_Te. (e) Comparison of power factor with different state-of-the-art SnTe-based TE systems: Sn_1–3*x*_In_*x*_Ag_2*x*_Te (*x* = 5%),^52^ Sn_0.96_Ga_0.07_Te,^95^ Cu_1.75_Te-SnTe,^42^ Bi– Zn co-doped SnTe,^96^ Mg–In co-doped SnTe,^97^ Sn_0.95_Ca_0.06_In_0.02_Te(Cu_2_Te)_0.05_,^40^ and Ca–In co-doped SnTe.^98^

Figures 4a and S9a display the electrical conductivity (σ) of both SnTe and SnTe-*y*% Cu_1.5_Te samples to monotonically decrease with temperature, as it corresponds with a degenerated semiconductor behavior. For SnTe, the electrical conductivity is about 3.4 × 10^5^ S m^–1^ at room temperature, which is consistent with our previous report.^73^ The electrical conductivity of all the composites is higher than that of SnTe in the whole temperature range, and it increases when increasing the Cu_1.5_Te concentration within the precursor. The increased electrical conductivity is in part associated with the presence of Cu within the SnTe structure, acting as an electron acceptor. At high enough Cu_1.5_Te concentrations, the formation of the Cu_2_SnTe_3_ phase may also contribute to the charge transport by accepting electrons or facilitating the charge transport at the grain boundary. Notice in this regard that Cu_2_SnTe_3_ is generally regarded as a low-carrier density semimetal.^88^ At the same time, the large grains generated in the presence of the Cu_2_SnTe_3_ phase also contribute to the electrical conductivity but have little effect on the Seebeck coefficient.^89^

Table S3 displays the Hall carrier concentration (η_H_) and mobility (μ_H_) of the different samples at room temperature. With increasing the precursor Cu_1.5_Te content, a clear increase of the charge carrier concentration and a simultaneous reduction of the mobility are obtained. η_H_ values increase almost an order of magnitude, from 3.2 × 10^20^ cm^–3^ for SnTe to 1.8 × 10^21^ cm^–3^ for SnTe–9% Cu_1.5_Te, while the mobility decreases a factor of 3, from 69.3 cm^2^ V^–1^ s^–1^ for SnTe to 27.8 cm^2^ V^–1^ s^–1^ for SnTe–9% Cu_1.5_Te.

Positive Seebeck coefficients monotonously increasing with temperature were obtained for all the samples in the whole temperature range measured, consistent with a p-type semiconductor behavior. *S* was higher for SnTe than for the different composites in the low temperature range, consistent with the charge carrier concentrations. However, in the highest temperature range tested, 700–800 K, the composite *S* exceeded that of pristine SnTe (Figures 4b and S9b).^42^ This reversal is attributed to the decreased Sn vacancies caused by the incorporation of Cu,^90^ whose anomalous behavior has been previously reported in SnTe-based TE materials.^42,43,74,91^Figure 4c displays the Pisarenko plot of the Seebeck coefficient as a function of the hole carrier concentration calculated using a two-valence-band model.^12^ The values obtained for all the samples follow the proper trend and are also consistent with previously reported data.

Overall, the power factors (PF, S^2^σ) of all the SnTe-*y*% Cu_1.5_Te composites were significantly larger than those of SnTe, especially in the medium–high temperature range tested, 600–800 K (Figures 4d and S9c). The highest power factors were obtained for the SnTe-7% Cu_1.5_Te composite, reaching 3.63 mW m^–1^ K^–2^ at 823 K, which is in the high-value range of the previously reported PF values for SnTe (Figure 4e and Table S4).

**Figure 5:**
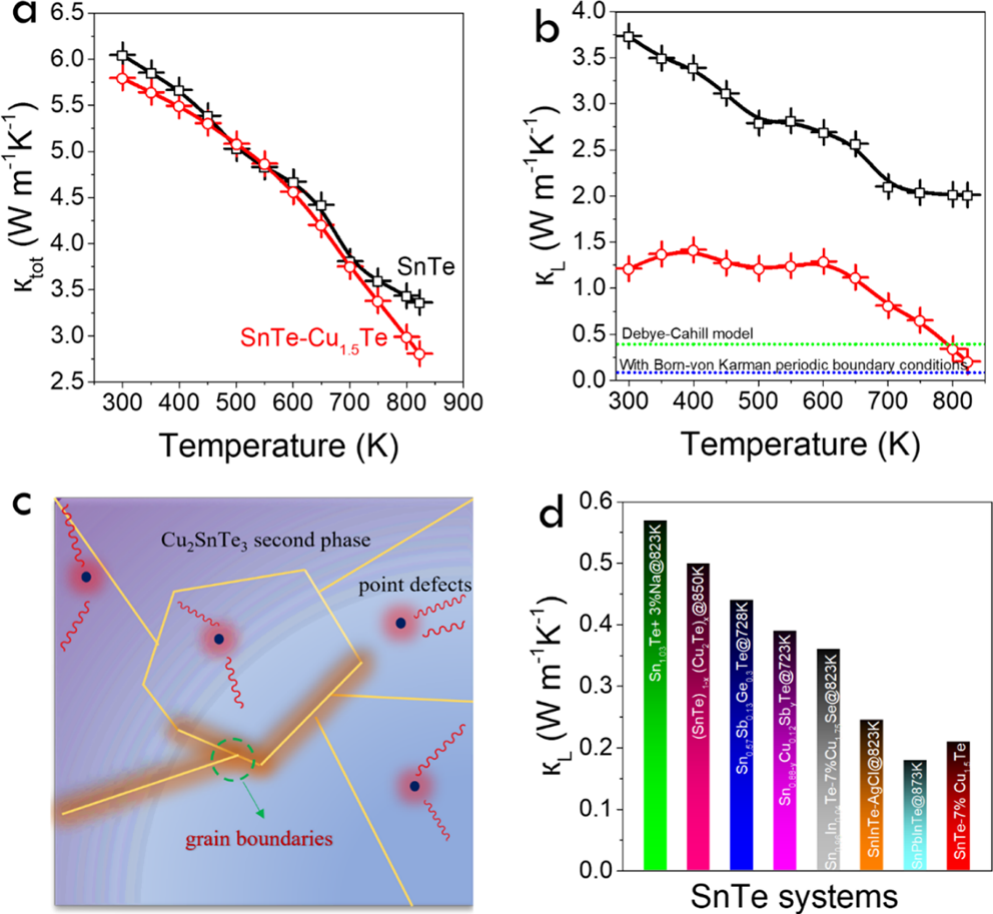
Temperature dependence of (a) total thermal conductivity, κ_total_; (b) lattice thermal conductivity, κ_L_, for SnTe and SnTe-7% Cu_1.5_Te. (c) Schematic diagram of various possible phonon scattering and κ_L_. (d) Comparison of the lowest κ_L_ over test temperature with previously reported SnTe-based systems: Sn_0.88–*y*_Cu_0.12_Sb_*y*_Te,^103^ Sn_0.85_Sb_0.15_Te,^104^ Sn_0.96_In_0.04_Te–7%Cu_1.75_Se,^34^ Sn_0.96_Pb_0.01_In_0.03_Te,^81^ (Sn_0.985_In_0.015_Te)_0.90_(AgCl)_0.10_,^55^ Sn_1.03_Te ^+^ 3% Na,^26^ Ag-doped SnTe,^54^ (SnTe)_1–*x*_(Cu_2_Te)_*x*_,^43^ and Sn_0.57_Sb_0.13_Ge_0.3_Te.^105^

As shown in Figures 5a and S9d, the total room temperature thermal conductivity (κ_tot_) of SnTe and the SnTe-*y*% Cu_1.5_Te composites was relatively high, around 6 W m^–1^ K^–1^. It monotonously decreased with temperature for all the samples, down to 3.4 W m^–1^ K^–1^ at 823 K for SnTe and 2.8 W m^–1^ K^–1^ for SnTe–7%Cu_1.5_Te. κ_tot_ decreased with the precursor Cu_1.5_Te content at small Cu_1.5_Te concentrations but returned toward the pristine SnTe values at the highest Cu_1.5_Te loadings tested. This complex evolution is related to the different contributions of the electron (κ_e_) and lattice (κ_L_) thermal conductivities. κ_e_ was calculated from the Wiedemann–Franz equation, κ_e_ = LσT, using the Lorenz number (*L*) estimated from the measured Seebeck coefficient, *L* = 1.5 + exp[−|*S*|/116] × 10^–8^.^99,100^ The obtained *L* values and κ_e_ can be found in Figure S9e,f. κ_L_ was evaluated by subtracting κ_e_ from κ_tot_ (Figure S9g). Significantly lower κ_L_ values were obtained for the composite materials compared with SnTe, and a clear decrease of κ_L_ was obtained when increasing the precursor Cu_1.5_Te content. The lowest κ_L_, 0.21 W m^–1^ K^–1^, was obtained for SnTe– 9% Cu_1.5_Te. This value was lower than the amorphous limit of SnTe as calculated from the Debye–Cahill model (≈0.4 W m^–1^ K^–1^).^101^ Meanwhile, this low κ_L_ was still higher than the Born-von Karman periodic boundary conditions (Figure 5b).^102^ The low κ_L_ obtained for the SnTe-*y*% Cu_1.5_Te composites is related to the multiscale scattering centers present in these samples, including the Cu impurities within the SnTe crystals, SnTe/Cu_2_SnTe_3_ nanoscale interphases, and SnTe/SnTe grain boundaries (Figure 5c). Notice that the compositional inhomogeneities can be particularly effective in scattering wide-frequency phonons.^55^ In this same direction, the slight lattice mismatch between the cubic SnTe (*a* = 6.303 Å) and Cu_2_SnTe_3_ (*a* = 6.047 Å) results in a lattice distortion that can introduce strain fields within the SnTe, further hampering phonon propagation.^53^ Besides, above 600 K, a clear decrease of κ_L_ is obtained for the samples containing the largest amounts of precursor Cu_1.5_Te, and it is associated with the melting of the Cu_2_SnTe_3_ phase at the grain boundaries. As can be seen from Figure 5d, SnTe-*y*% Cu_1.5_Te composites are characterized by relatively low κ_L_ values compared with previous reports. Similar ultralow κ_L_ values, down to 0.18 W m^–1^ K^–1^, were achieved for hydrothermally synthesized Sn_0.96_Pb_0.01_In_0.03_Te samples and were associated with reduced grain sizes, scattering by nanoparticles and point defects.^81^

**Figure 6:**
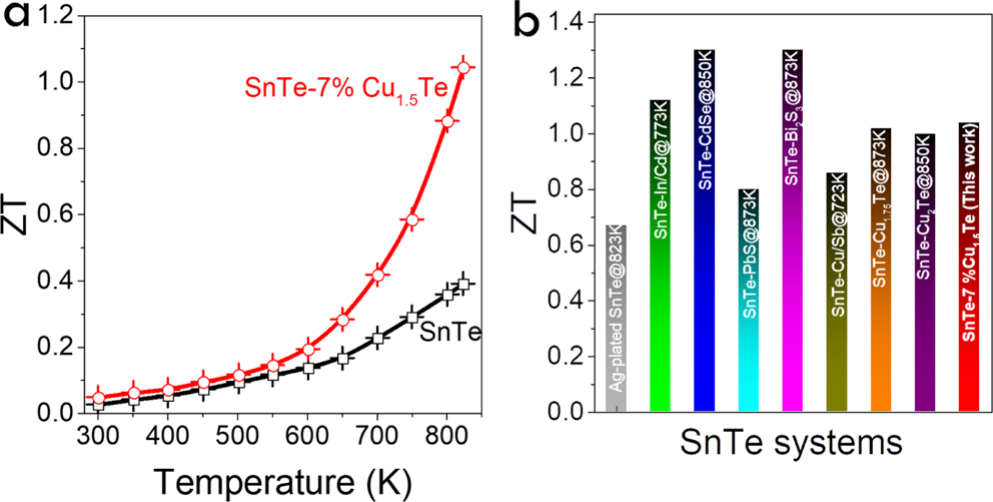
Temperature dependence of (a) TE figure of merit ZT values for SnTe-7% Cu_1.5_Te. (b) Comparison of peak ZT values for SnTe-based TE materials in this work and previous reports, including solution-based preparation such as Ag,^54^ In/Cd,^82^ CdSe,^73^ PbS,^74^ Bi_2_S_3_,^106^ Sb_2_Se_3_,^94^ and Cu-incorporation solid phase sintering of Cu/Sb,^103^ Cu_1.75_Te,^42^ and Cu_2_Te.^43^

Overall, the enhanced PF and reduced κ_L_ obtained for the SnTe-*y*% Cu_1.5_Te composites resulted in significantly increased ZT values with respect to SnTe, particularly in the medium/high-temperature range, reaching a maximum of 1.04 at 823 K for the SnTe–7% Cu_1.5_Te nanocomposite (Figures 6a and S9h).^82^ This high ZT represents a 170% enhancement compared with pristine SnTe. Figure 6b shows a comparison of the ZT values obtained for the SnTe-7% Cu_1.5_Te at 823 K with those previously reported for SnTe-based materials, including solution-based preparation, such as Ag,^54^ In/Cd,^82^ CdSe,^73^ PbS,^74^ Bi_2_S_3_,^106^ Sb_2_Se_3_,^94^ and Cu-incorporation solid phase sintering of Cu/Sb,^103^ Cu_1.75_Te,^42^ and Cu_2_Te.^43^ The composites reported here display relatively large ZT values in this temperature range. The ZT values of SnTe-based TE materials reported in recent years are summarized in Table S4. As shown in Figure S10, measurements from multiple samples confirmed the reproducibility of the obtained values. Besides, thermal gravimetric analysis (TGA) of the composites confirmed their excellent thermal stability against a loss of chalcogen or chalcogenide phases (Figure S11). Actually, compared with the pristine SnTe sample, the SnTe-7% Cu_1.5_Te composite exhibits enhanced stability against the slight volatilization of Sn observed in SnTe.^51^ On the other hand, the SnTe-7% Cu_1.5_Te composite presented a slightly lower hardness than the SnTe sample (Figure S12).

## Conclusions

In summary, a Sn–Te inorganic molecular precursor ink was prepared by dissolving a Sn salt and Te in a hydrazine- and thiol-free solvent system. The thermal decomposition of this precursor at 280 °C resulted in the formation of pure SnTe. The SnTe precursor ink was combined with a colloidal suspension of Cu_1.5_Te nanocrystals to produce SnTe–Cu_2_SnTe_3_ nanocomposites. The presence of Cu_1.5_Te in the precursor solution enhanced the growth of the SnTe crystal domains due to the formation of the low melting point phase Cu_2_SnTe_3_. The SnTe–Cu_2_SnTe_3_ nanocomposites showed significantly larger electrical conductivities than SnTe, which was in part related to the Cu doping with the SnTe crystals acting as acceptors. At the same time, slightly larger Seebeck coefficients were also obtained with the introduction of Cu_1.5_Te within the precursor solution, particularly in the medium/high-temperature range. Thus, overall higher PFs up to 3.63 mW m^–1^ K^–2^ were obtained for the SnTe–Cu_2_SnTe_3_ composites. Besides, the presence of Cu ions within the SnTe crystal and the abundant Cu_2_SnTe_3_ nanoprecipitates significantly reduced the lattice thermal conductivity, down to 0.21 W m^–1^ K^–1^. Ultimately, a ZT value of 1.04 was achieved at 823 K due to the simultaneous improvement of electrical and thermal transport properties. The strategy reported here not only provides an alternative approach for the preparation of functional metal chalcogenides and related nanocomposites from easily processable and scalable molecular precursor inks but also provides a meaningful perspective for developing high-performance lead-free medium/high-temperature TE materials.

## Supporting Information Available

Experimental details; comparison of performance; and additional SEM, XRD, EDX, HRTEM, TGA, hardness, and transport property characterization (PDF)
