# Supplementary material for: Bottom-Up Synthesis of SnTe-Based Thermoelectric Composites
Source: ACS Appl Mater Interfaces. 2023 May 4;15(19):23380–9. doi: 10.1021/acsami.3c00625 (PMC10197086; doi:10.1021/acsami.3c00625)
Supplement: Supplementary file 1 — am3c00625_si_001.pdf [file am3c00625_si_001.pdf]

Supporting Information

Bottom-up Synthesis of SnTe-based Thermoelectric Composites

Bingfei Nan [a, b], Xuan Song [c], Cheng Chang [d, e], Ke Xiao [a, b], Yu Zhang [f], Linlin Yang [a, b], Sharona Horta [d], Junshan Li [g], Khak Ho Lim [h, i], Maria Ibáñez [d,*], and Andreu Cabot [a, j,*]

[a] Catalonia Institute for Energy Research—IREC, Sant Adrià de Besòs, Barcelona 08930, Spain

[b] Universitat de Barcelona, Martí i Franquès 1, 08028, Barcelona, Spain

[c] The State Key Laboratory of Chemical Engineering, Department of Chemical Engineering, Tsinghua University, Beijing 100084, China

[d] Institute of Science and Technology Austria, Am Campus 1, 3400 Klosterneuburg, Austria

[e] School of Materials Science and Engineering, Beihang University, Beijing 100191, China.

[f] Department of Materials Science and Engineering, Pennsylvania State University, University Park, Pennsylvania 16802, United States.

[g] Institute of Advanced Study, Chengdu University, Chengdu 610106, China

[h] Institute of Zhejiang University- Quzhou, 99 Zheda Rd, Quzhou 324000, Zhejiang, P.R. China

[i] College of Chemical and Biological Engineering, Zhejiang University, 38 Zheda Rd, Hangzhou 310007, Zhejiang, P.R. China

[j] ICREA, Pg. Lluís Companys 23, 08010 Barcelona, Catalonia, Spain

* E-mail addresses: mibanez@ist.ac.at (M. Ibáñez), acabot@irec.cat (A. Cabot).

EXPERIMENTAL SECTION

Chemicals and materials. Tin(II) chloride anhydrous ($SnCl_2$, 99%), tri-n-octylphosphine (TOP, $C_{24}H_{51}P$, 90%), and oleylamine (OAm, $C_{18}H_{37}N$, approximate C18-content 80-90%) were purchased from the Fisher. Copper(II) acetate monohydrate ($Cu(AC)_2 \cdot H_2O$, ≥98%) and tellurium (Te, shot 1-2 mm, 99.999%) were ordered from Sigma-Aldrich. All solvents including hexane and ethanol were obtained from various sources. OAm was heated to 120 °C under vacuum for 120 min to remove residues. The other chemicals were directly used as received, without any further purification.

Preparation of tri-n-octylphosphine telluride (TOPTe): Considering the long time required to completely react tellurium shots with TOP, a 2M tellurium precursor stock solution (TOPTe) was first prepared within an Ar-filled glovebox. In detail, 200 mmol of tellurium powder was dissolved into 100 ml of TOP by vigorous stirring and heating at 60 °C. According to our experience, it takes about 5-7 days to yield a 2 M completely dissolved yellowish TOPTe. The prepared TOPTe was kept within glovebox until further use.

Sn-Te precursor ink and SnTe synthesis. A $SnCl_2$-OAm solution was prepared by dissolving 4 mmol of $SnCl_2$ into 30 mL of OAm under air atmosphere. The solution was sonicated until the complete formation of a light-yellow solution. Then, 2mL of the 2M TOPTe precursor solution was injected into the $SnCl_2$-OAm solution to form a $SnCl_2$/TOPTe-OAm ink solution. Upon TOPTe injection, the color of the solution gradually changed from dark yellow to dark brown within several minutes. The $SnCl_2$/TOPTe-OAm solution was then degassed at room temperature for 20 min and further vacuumed at 120 ºC for 20 min with magnetic stirring. Subsequently, the mixture was heated to 280 ºC at a rate of ~10 ºC /min and reacted at this temperature for 60 min under Ar atmosphere. When the reaction was completed, the solution was cooled down to room temperature in a water bath. The harvested crude solution containing chloroform was centrifuged at 9000 rpm for 5 min. The re-dispersed precipitate using a mixture solvent of chloroform and ethanol ($V_{chloroform}$: $V_{ethanol}$=1:2) was washed and centrifuged more than one time at 8000 rpm for 3 min. The fine product was dried in a vacuum and stored in a glovebox for further use.

Synthesis of copper telluride nanocrystals ($Cu_{1.5}Te$ NCs). $Cu_{1.5}Te$ NCs were synthesized by

a colloidal method reported from our group with minor modifications.[1] In a typical synthesis, 10 mmol of Cu $(AC)_2$ was mixed with 50 mL of OAm in a 100 mL three-neck flask. The mixture was kept under vacuum for 20 min at room temperature, then heated to 120 ºC and maintained at this temperature for 30 min under vacuum to remove low boiling point impurities. Then the temperature was increased to 220 ºC under Ar. After ~10 min, the initial bright yellow solution became clear light brown. At this point, 10 mL of 1 M TOPTe was injected, and the light brown solution immediately changed color to deep green. Upon injection, the temperature of the reaction mixture dropped to ~210 ◦C. The mixture was allowed to recover the 220 ºC and maintained at this point for 30 min. Afterwards, the colloidal solution was rapidly cooled to room temperature with a water bath at an initial approximate rate of ~80 ◦C/min. Finally, 25 mL of chloroform were added to the crude solution and the mixture was sonicated for several minutes. The final deep green product was precipitated by centrifugation at 8000 rpm for 5 min. Then it was redispersed in chloroform and precipitated one more time by centrifugation in the presence of ethanol. Finally, $Cu_{1.5}Te$ NCs were redispersed in chloroform and kept in an Ar-filled glovebox for further use.

SnTe-$Cu_{1.5}$Te precursor. Briefly, to prepare a SnTe-$Cu_{1.5}$Te precursor solution, a certain amount mass fraction of $Cu_{1.5}Te$ nanocrystals dispersed in OAm (10 mg·mL^{-1}) were added into the as-prepared SnTe precursor ink. After a period of intense sonication and stirring, SnTe-$Cu_{1.5}$Te precursor solutions were formed. This precursor was thermally decomposed following the same procedure as described above for the SnTe NCs.

Annealing and hot-press. A certain amount of the dried nanopowders was heated to 580 °C at a rate of 10 °C/min and maintained for 120 min under Ar atmosphere. The annealed powders were loaded into a graphite die within the glovebox containing Ar gas, and then compressed and sintered into pellets by using home-made hot-pressing machine under the axial compressive stress of 40 MPa at 500 °C for 5 min. Finally, dense disk-shaped pellets with diameter of approximate Φ10 mm were obtained, which were then stored in a glovebox and polished before further measurements.

Material characterizations. SEM analysis was done in a Zeiss. Size and shape of the NCs were analyzed by transmission electron microscopy (TEM) using a ZEISS LIBRA 120

instrument, operating at 120 kV. X-ray powder diffraction (XRD) were performed on a Bruker AXS D8 Advance X-ray diffractometer with Ni-filtered (2 mm thickness) Cu Kα radiation (λ=1.5406 Å) operating at 40 kV and 40mA to identify phase and structure of samples. X-ray photoelectron spectroscopy (XPS) Analysis was performed on a SPECS system. Thermogravimetric analysis (TGA) was carried out on a Netzsch DSC at a heating rate of 10 K min^{-1} with argon gas atmosphere.

TE performance measurement. The hot-pressed sintered disk samples were polished with abrasive paper. The pellets are approximately10 mm in diameter and 1-1.5 mm in thickness. Seebeck coefficient and electrical resistivity were simultaneously measured on LSR-3 Linseis system under helium atmosphere. Electrical properties were obtained by measuring the samples resistivity by the standard four-point probe method. The thermal diffusivity (λ) was measured on LFA 1000 Laser Flash (Netzsch, Germanny), and the total thermal conductivity (κ_{tot}) was calculated by the equation $\kappa_{\text{tot}} = \lambda\ C_p\ \rho$. C_p is the specific heat capacity, which was determined by the Dulong-Petit law.[2] ρ is the density of samples, and it was calculated via the Archimedes drainage method. For evaluation of carrier concentration (n_H) and mobility (μ_H) at room temperature, Hall coefficient measurement system were performed on a Hall Effect Analyzer by Linseis Company with a magnetic field of 0.6 T (ezHEMS, NanoMagnetics) to obtain these two parameters via $n_H = 1/eR_H$ and $\mu_H = \sigma R_H$, respectively, where e is the electronic charge, R_H is Hall coefficient.

Thermal conductivity (κ) and Lorenz number (L) calculation:

The total thermal conductivity (κ_{tot}) usually includes the electronic thermal conductivity (κ_e) and lattice thermal conductivity (κ_L).

$$\kappa_{\text{tot}} = \kappa_{\text{e}} + \kappa_L \quad (1)$$

The κ_e is proportional to the electrical conductivity σ according to the Wiedemann-Franz relation (equation 2).

$$\kappa_{\text{e}} = L\sigma T \quad (2)$$

L can be calculated considering a single parabolic band (SPB) model via the Fermi integral function[3, 4]

$$L = \left(\frac{k_B}{e}\right)\left(\frac{\left(r+\frac{7}{2}\right)F_{r+5/2}(\eta)}{\left(r+\frac{3}{2}\right)F_{r+1/2}(\eta)} - \left[\frac{\left(r+\frac{5}{2}\right)F_{r+3/2}(\eta)}{\left(r+\frac{3}{2}\right)F_{r+1/2}(\eta)}\right]^2\right) \quad (3)$$

Here, k_B is the Boltzmann constant. e is the electron charge. h is the Planck constant. The scattering factor r is assumed to be $-\frac{1}{2}$.

The reduced Fermi level η is a dimensionless parameter that corresponds one-to-one to the Seebeck coefficient S.[5] Thus, the calculation of η can be derived from the measured S by using the following relationship

$$S = \pm\frac{k_B}{e}\left(\frac{\left(r+\frac{5}{2}\right)F_{r+3/2}(\eta)}{\left(r+\frac{3}{2}\right)F_{r+1/2}(\eta)} - \eta\right) \quad (4)$$

$F_n(\eta)$ is the nth order Fermi integral,

$$F_n(\eta) = \int_0^\infty \frac{\zeta^n}{1+e^{\zeta-\eta}}d\zeta \quad (5)$$

L can be obtained by combining equations (3), (4) and (5). Some standard values used in these calculations are shown in Table S5. The simplified L is widely used: [6]

$$\mathrm{L} = 1.5 + \exp\left[-\frac{|\mathrm{S}|}{116}\right] \times 10^{-8}$$

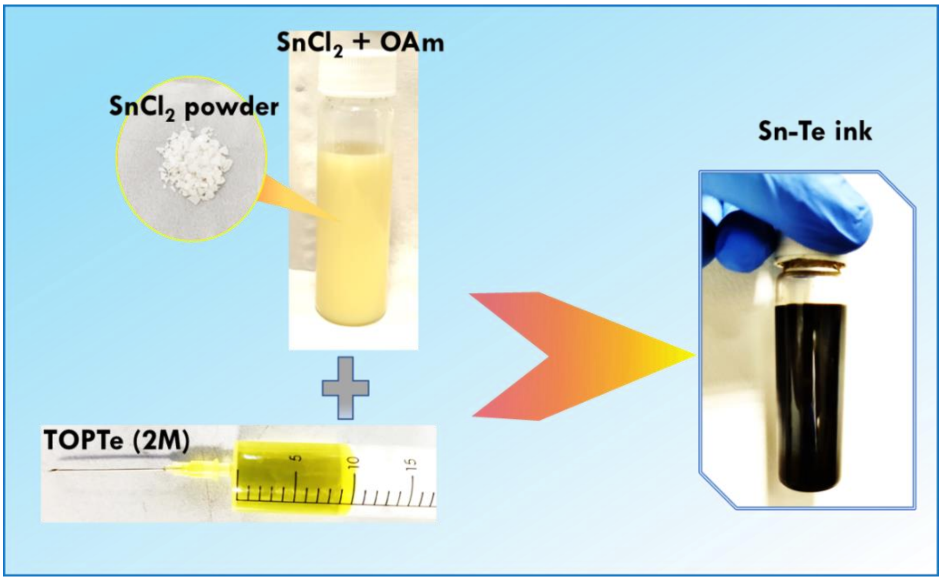

Figure S1. Illustration of Sn-Te ink obtained from $SnCl_2$ powder, TOPTe, and OAm.

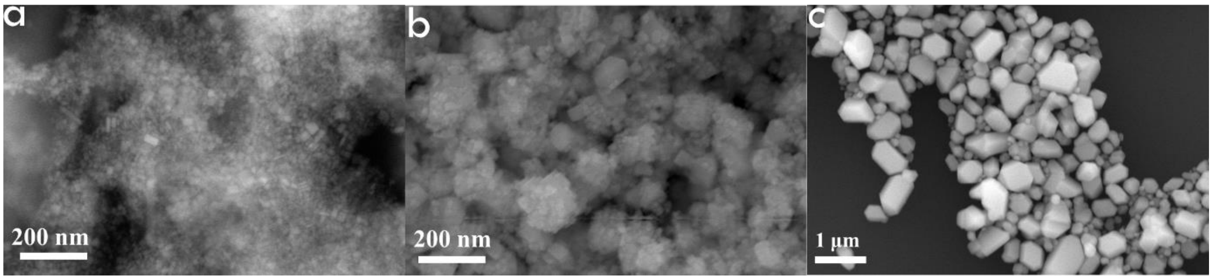

Figure S2. SEM images of SnTe obtained at various temperatures: (a) 200 ºC, (b) 240 ºC and (c) 280 ºC.

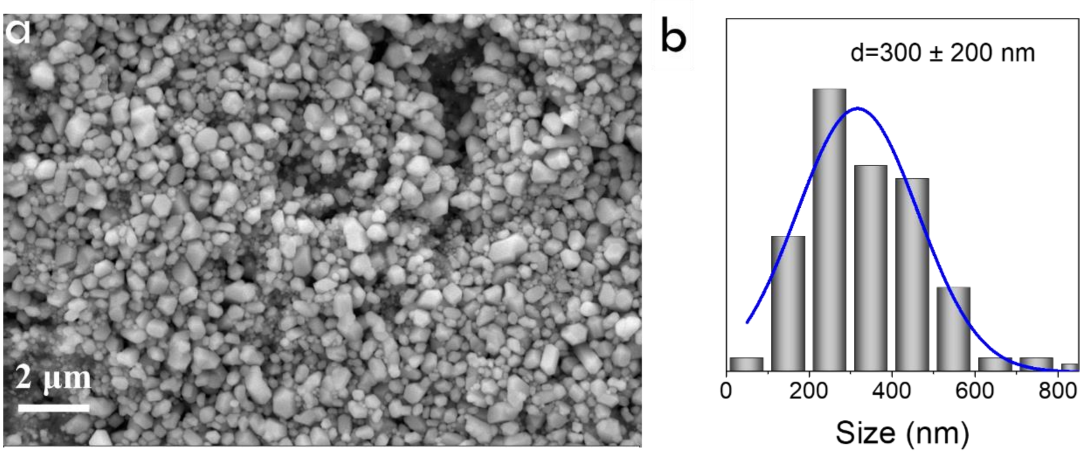

Figure S3. (a) SEM image and (b) particles size of SnTe NCs obtained at 280 ºC.

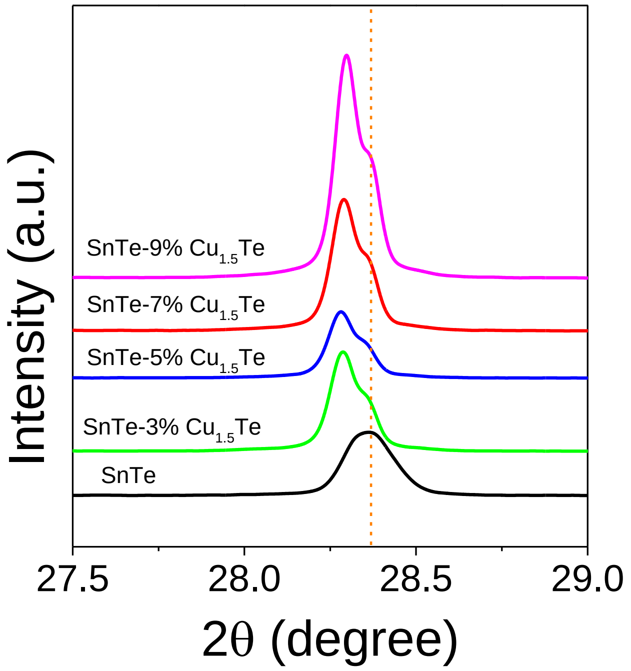

Figure S4. SnTe (200) XRD peak of the different SnTe-y%$Cu_{1.5}Te$ composites.

Samples	Cross-section SEM	EDX maps
SnTe-3% $Cu_{1.5}Te$	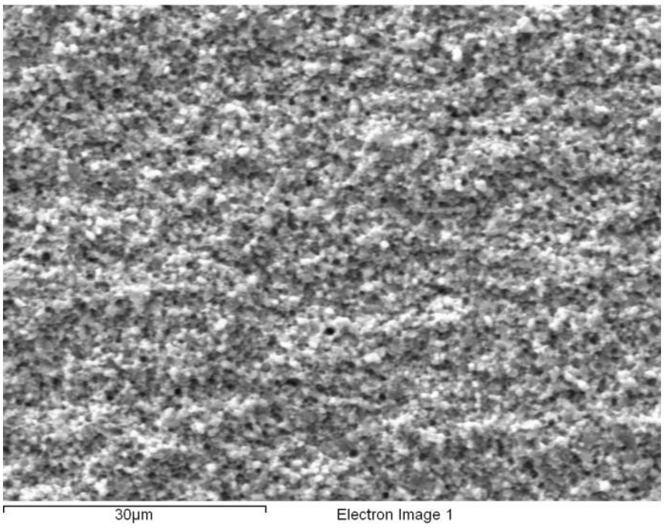	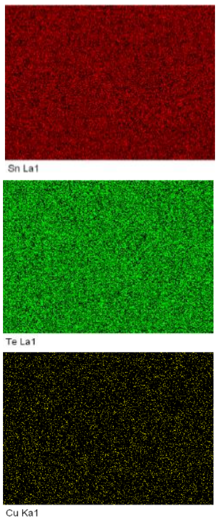
SnTe-5% $Cu_{1.5}Te$	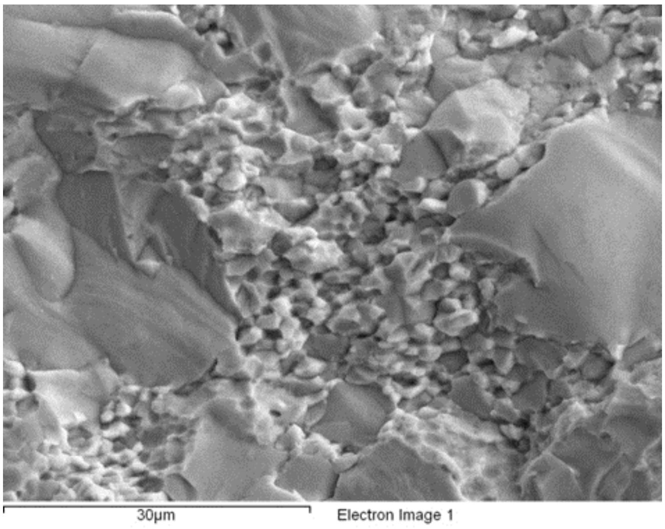	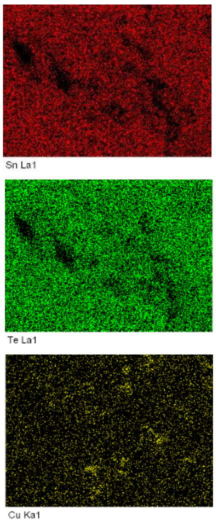
SnTe-9% $Cu_{1.5}Te$	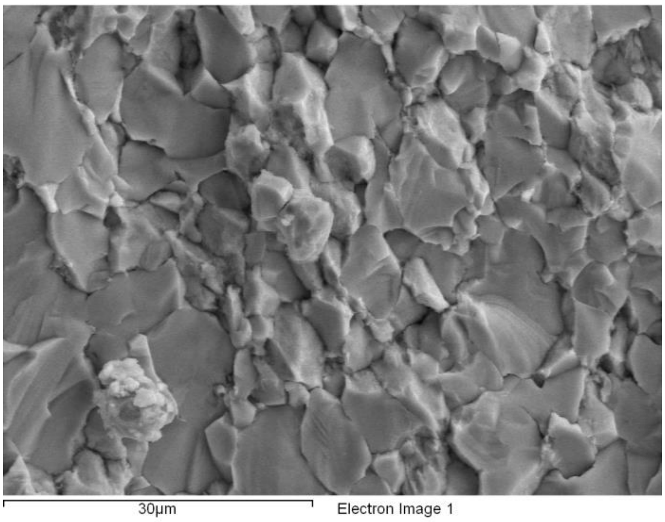	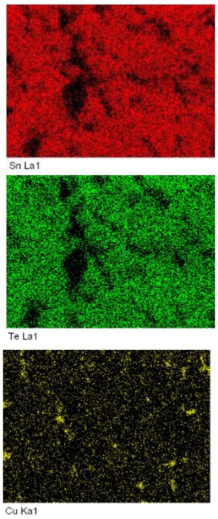

Figure S5. Cross-section SEM-EDX maps for various SnTe-y% $Cu_{1.5}Te$ pellet.

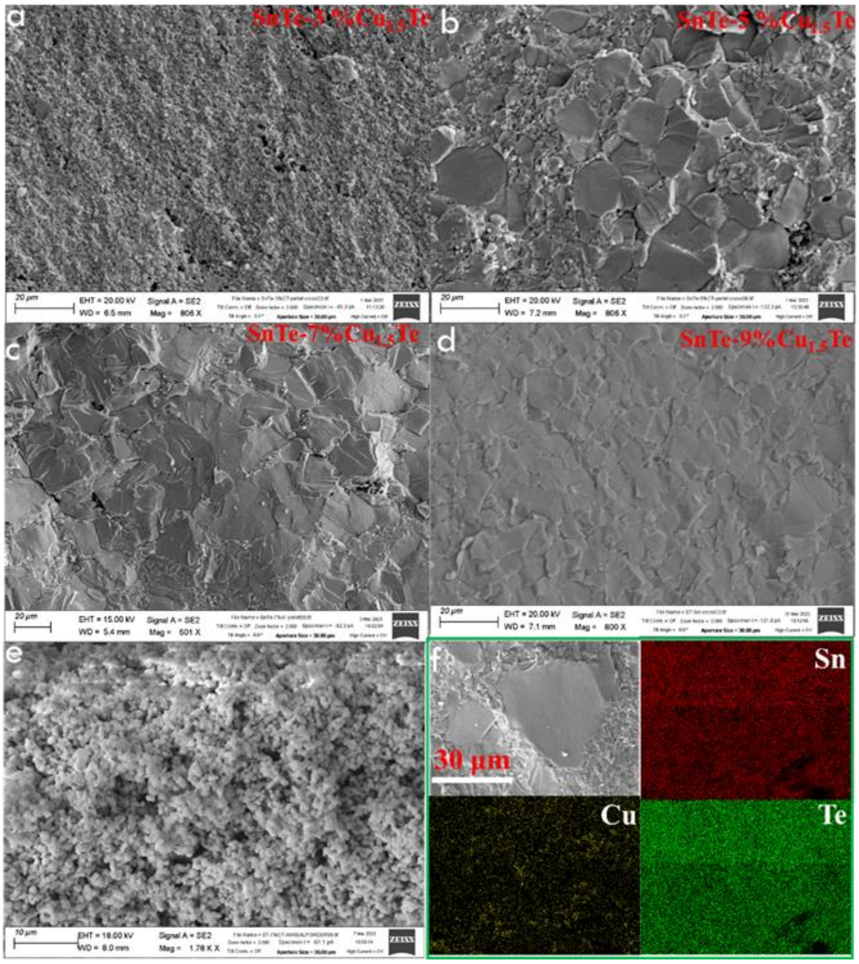

Figure S6. Cross-section SEM images (a-d) for various SnTe-y% $Cu_{1.5}$Te pellet (y=3, 5, 7 and 9). (e) SEM annealed powder of SnTe-7%$Cu_{1.5}$Te. (f) Cross-section SEM image of the sintered SnTe-7%$Cu_{1.5}$Te grains from the pellet and corresponding EDX maps of Sn, Te, and Cu.

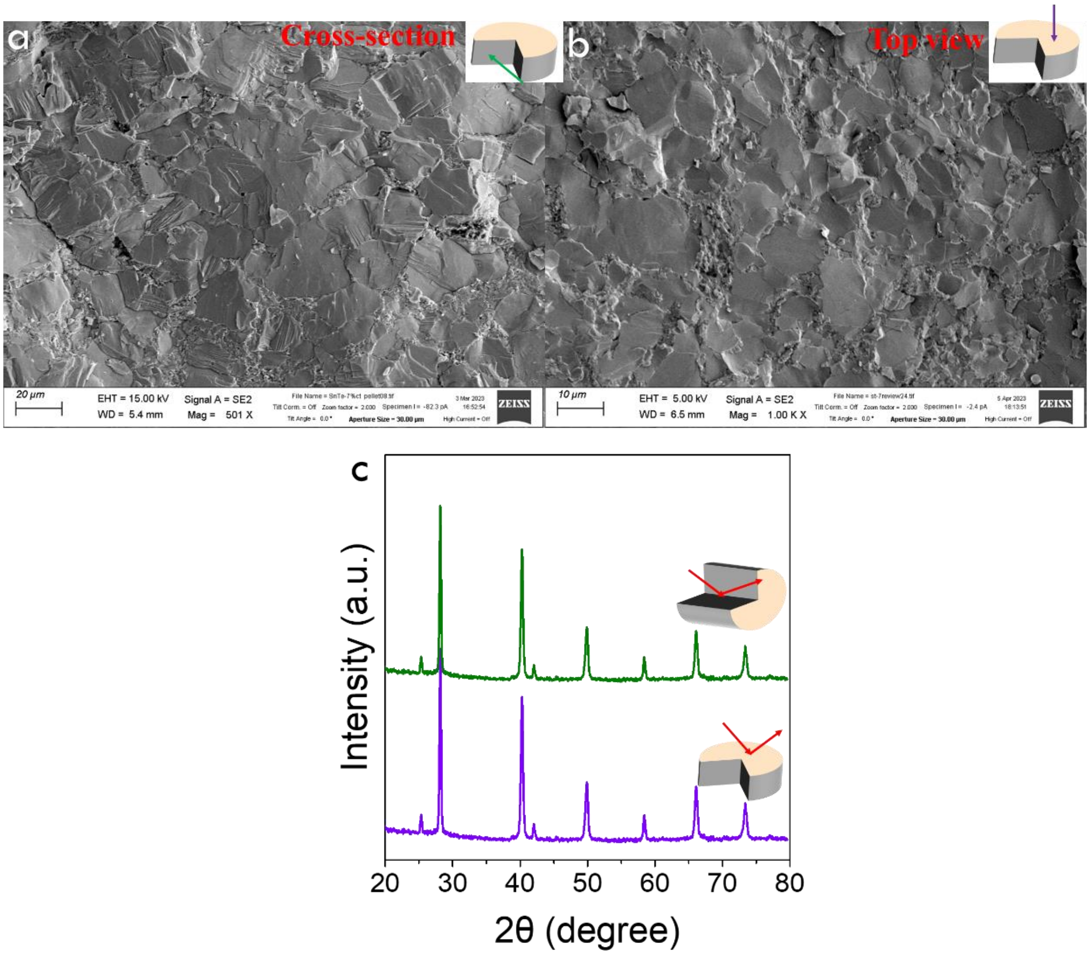

Figure S7. (a) and (b) SEM images, and (c) XRD patterns of SnTe-7% $Cu_{1.5}Te$ pellets in two normal directions.

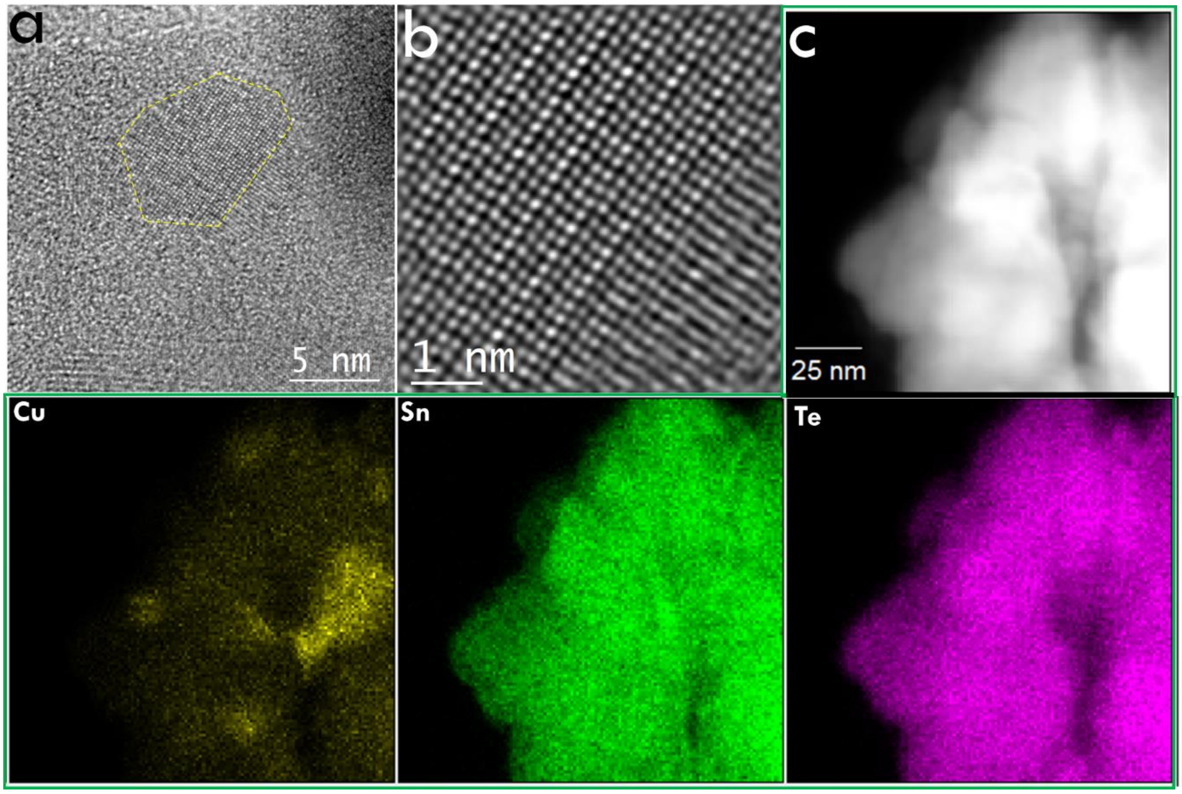

Figure S8. HRTEM image of a SnTe-7% $Cu_{1.5}Te$ composite. (a) HRTEM image of the main SnTe matrix and embedded Cu_2SnTe_3 nanodomain within the yellow dashed area. (b) Enlarged HRTEM image of the Cu_2SnTe_3 nanodomain. (c) HAADF image and corresponding EDX maps of Cu, Sn and Te areas.

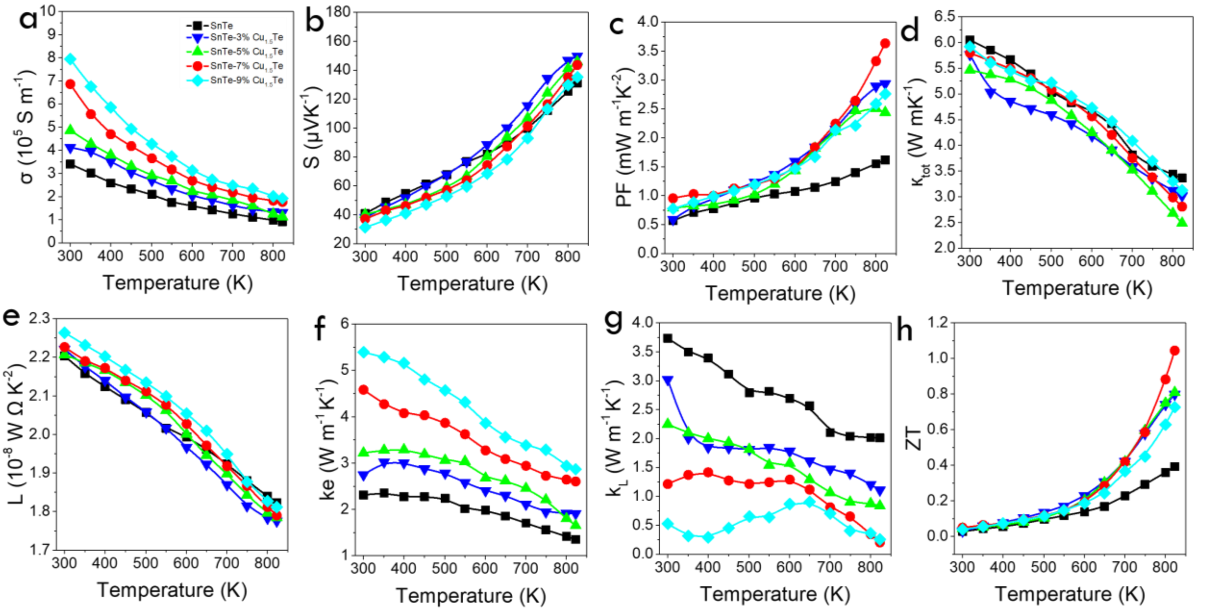

Figure S9. Temperature dependence of SnTe-y%$Cu_{1.5}$Te composites. (a) electrical conductivity; (b) Seebeck coefficient; (c) power factor; (d) total thermal conductivity; (e) Lorenz number; (f) electronic thermal conductivity; (g) lattice thermal conductivity; (h) ZT.

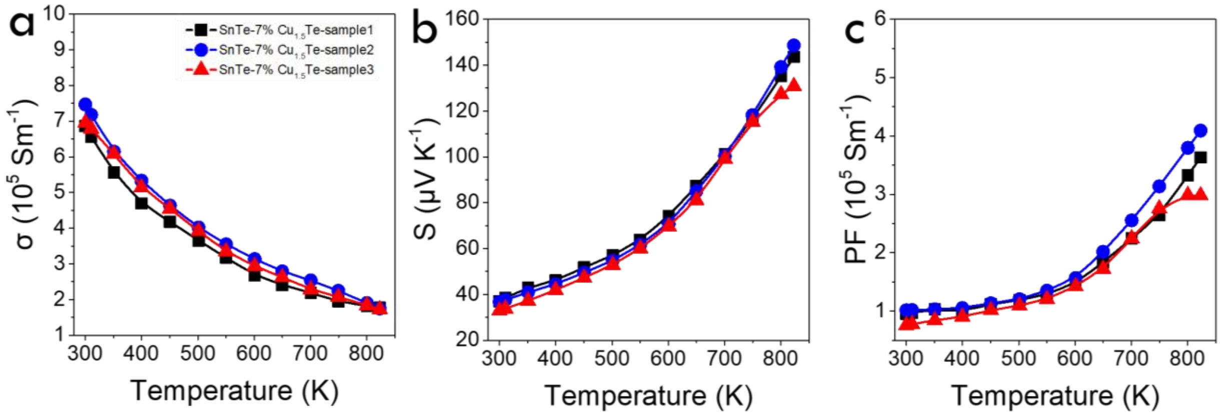

Figure S10. Temperature dependence of (a) electrical conductivity; (b) seebeck coefficient; (c) PF of three different SnTe-7% $Cu_{1.5}$Te pellets.

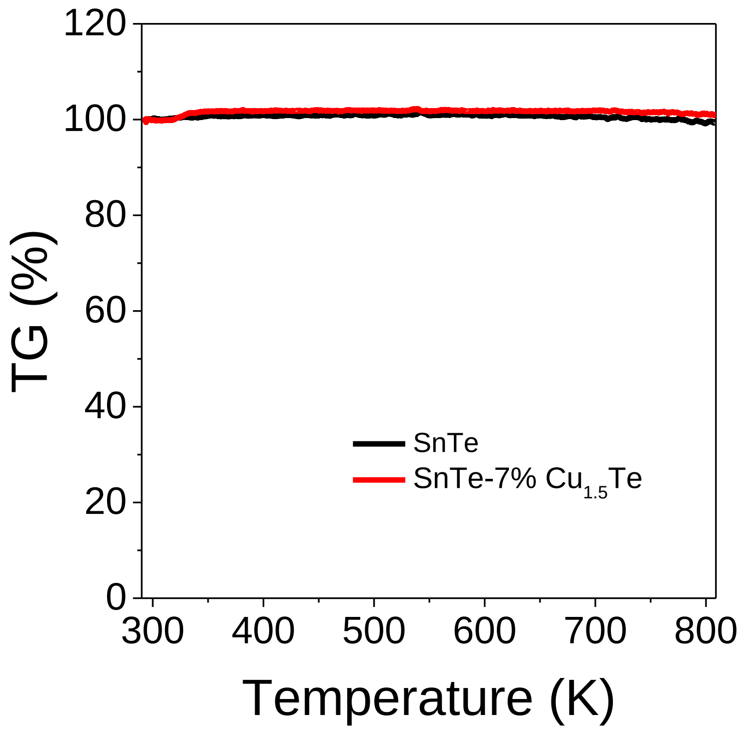

Figure S11. Thermal gravimetric analysis (TGA) results of SnTe and SnTe-7% $Cu_{1.5}Te$ samples.

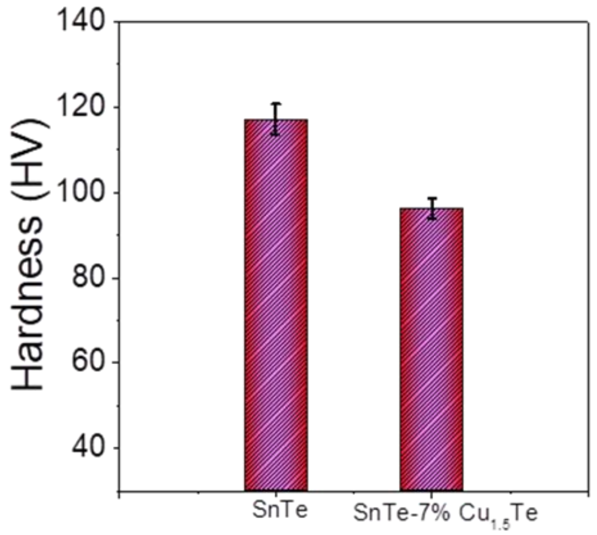

Figure S12. Hardness of the SnTe and SnTe-7% $Cu_{1.5}Te$ samples

Table S1. The atomic ratios of as-synthesized SnTe and SnTe-y%$Cu_{1.5}Te$ powders obtained from SEM-EDX analysis.

Samples \ Elements	Sn	Te	Cu
SnTe	52.3	47.8	0
SnTe-3% $Cu_{1.5}Te$	50.8	47.2	2.0
SnTe-5% $Cu_{1.5}Te$	48.6	48.4	3.0
SnTe-7% $Cu_{1.5}Te$	47.9	47.9	4.2
SnTe-9% $Cu_{1.5}Te$	47.6	47.2	5.2

Table S2. Densities of SnTe-y%$Cu_{1.5}Te$ samples determined by the Archimedes method.

Sample	Actual Density (g cm^{-3})	Relative Density (%)
SnTe	6.36	97.7
SnTe-3% $Cu_{1.5}Te$	6.41	98.5
SnTe-5% $Cu_{1.5}Te$	6.37	97.8
SnTe-7% $Cu_{1.5}Te$	6.38	98.0
SnTe-9% $Cu_{1.5}Te$	6.45	99.1

Table S3. Room temperature Hall charge carrier concentration and mobility for the SnTe-y%$Cu_{1.5}Te$ samples.

Sample	η_H (cm^{-3})	μ_H (cm^2 V^{-1} s^{-1})
SnTe	3.2×10^{20}	69.3
SnTe-3% $Cu_{1.5}Te$	4.4×10^{20}	60.5
SnTe-5% $Cu_{1.5}Te$	8.5×10^{20}	34.4
SnTe-7% $Cu_{1.5}Te$	1.5×10^{21}	28.7
SnTe-9% $Cu_{1.5}Te$	1.8×10^{21}	27.8

Table S4. Partial progress of SnTe-based thermoelectric materials in recent years.

Dopant	Method	PF ($mW\ m^{-1}\ K^{-2}$)	peak ZT@T	Year refs
Ge/Pb/Sb/Mn	SHS-HG-SPS	1.72	~ 1.1@873 K	2023 [7]
Ge/Bi/$AgBiTe_2$	M+SPS	2.58	1.45@873 K	2023 [8]
$CuSbSe_2$	M+HP	2.361	1.1@823 K	2023 [9]
Pb/In/Se	M+SPS	~1.996	~0.86@773 K	2023 [10]
Cd/Sb/Cu_2Se	M+HP	2.421	1.52@833 K	2023 [11]
In/Ag_2S	M+SPS	1.92	1.02@800 K	2023 [12]
Ge/Bi/Sb	M+HP	~2.57	~1.1@873 K	2023 [13]
Sb_2Te_3/Ge	M+HP	~2.5	~1.3@723 K	2023 [14]
Gd	MA+SPS	2.922	1.5@900 K	2022 [15]
Sb/$GeMnTe_2$	M+HP	2.28	1.4@850 K	2022 [16]
Mn/$BiBr_3$	M+SPS	2.385	~1.31@873 K	2022 [17]
MnTe/Cu_2Te	M+SPS	2.5	1.3@873 K	2022 [18]
In/AgCuTe	M+SPS	2.14	1.14@800 K	2022 [19]
Mn/$AgSbTe_2$	M+SPS	2.81	1.8@873 K	2022 [20]
Ge/$Ag_{0.5}Bi_{0.5}Se$/ZnO	M+SPS	2.25	~1.2@870 K	2022 [21]
Cu/Sb	M+HP	~2.2	0.86@723 K	2022 [22]
Na	M+SPS	3.793	1.26@898 K	2022 [23]
Mn-Bi	M+HP	2.22	1.3@850 K	2021 [24]
Sb	SM+SPS	2.32	~1.08@873 K	2021 [25]
Ag/Mg	M	2.7	1.55@865 K	2021 [26]
Mn/Bi	M+HP	~2.43	0.93@773 K	2021 [27]
$CuInTe_2$/SnTe	CS+HP	2.1	1.68@823 K	2021 [28]
Pb/$AgSbTe_2$	M+SPS	~2.55	~1.1@823 K	2021 [29]
Ag/Y	M+SPS	~2.485	~1.2@873 K	2021 [30]
Bi/Zn	M+HP	~3.6	~1.6@840 K	2020 [31]
PbTe	M+HP	~3.458	~1.2@750 K	2020 [32]
Pb/Zn	M+HP	~3.04	1.66@840 K	2020 [33]
Ge/Sb/Cu_2Te	M+SPS	3	1.5@873 K	2020 [34]
Pb/In	SM+SPS	~2.04	~0.7@873 K	2020 [3]
In/Ag	SM+SPS	~3.567	~1.38@823 K	2020 [35]
In/AgCl	MW+ SPS	2.64	0.86@823 K	2020 [36]
$AgBiSe_2$	M+HP	2.21	1.02@860 K	2020 [37]
CdSe	CS+HP	1.3	1.3@850 K	2019 [38]
In/$Cu_{1.75}Se$	M+SPS	~2.4	1.7@823 K	2019 [39]
In/Sr	SM+SPS	~3.388	~1.31@823 K	2019 [40]
In	SM+SPS	~2.18	~0.78@773 K	2019 [41]
Ca/In	SHS+SPS	~4.7	~1.65@840 K	2018 [42]
In/MgAgSb	M+SPS	1.767	1.41@835 K	2018 [43]

In/Cd	SM+ SPS	~2.676	1.12@773 K	2018 [44]
Mg/In	SHS+SPS	~4.2	1.5@840 K	2017 [45]
Se/Cd	MW+SPS	~2.31	~0.78@773 K	2017 [2]
Ga	M+HP	2.6	~1@873 K	2017 [46]
CaTe	M+HP	2.6	1.35@873 K	2016 [47]
Ag/In	M	~3.14	1@ 856 K	2016 [48]
In_2Te_3	M+SPS	2.37	~1.1@923 K	2015 [49]
Mn	M+SPS	2.3	1.3@900 K	2015 [50]
Mg	M+SPS	~3.03	~1.2@860 K	2015 [51]
$AgBiTe_2$	M+SPS	2.3	~1.1@775 K	2014 [52]

Note:

SHS-HG: self-propagating high-temperature synthesis under high-gravity field; M: raw materials are sealed into tubes and heated to melt; MA: mechanical alloying; SM: solvothermal method; MW: microwave-stimulated wet-chemical method; CS: colloidal synthesis; SHS: self-propagating high temperature synthesis process; SPS: spark plasma sintering; HP: hot-pressing method. Some of PF values were read from refs, not raw data provided by authors.

Table S5. Standard values used in the calculations [23].

Parameter	Symbol	Value
Boltzmann constant	k_B	1.38×10^{-23} J K^{-1}
Plank constant	h	1.0546×10^{-34} J s
Scattering factor	r	$-\frac{1}{2}$
Electron charge	e	1.602×10^{-19} C

REFERENCES

(1) Zhang, Y.; Xing, C.; Liu, Y.; Li, M.; Xiao, K.; Guardia, P.; Lee, S.; Han, X.; Ostovari Moghaddam, A.; Josep Roa, J.; Arbiol, J.; Ibáñez, M.; Pan, K.; Prato, M.; Xie, Y.; Cabot, A. Influence of copper telluride nanodomains on the transport properties of n-type bismuth telluride. *Chem. Eng. J.* **2021**, *418*, 129374.

(2) Wang, L.; Chang, S.; Zheng, S.; Fang, T.; Cui, W.; Bai, P.-p.; Yue, L.; Chen, Z.-G. Thermoelectric Performance of Se/Cd Codoped SnTe via Microwave Solvothermal Method. *ACS Appl. Mater. Interfaces* **2017**, *9*, 22612-22619.

(3) Lu, W.; He, T.; Li, S.; Zuo, X.; Zheng, Y.; Lou, X.; Zhang, J.; Li, D.; Liu, J.; Tang, G.

Thermoelectric performance of nanostructured In/Pb codoped SnTe with band convergence and resonant level prepared via a green and facile hydrothermal method. *Nanoscale* **2020**, *12*, 5857-5865.
(4) Hu, Z.; Xu, H.; Yan, C.; Liu, Y.; Han, Q.; Cheng, L.; Li, Z.; Song, J. Enhancement of the Thermoelectric Performance of Cu_2GeSe_3 via Isoelectronic (Ag, S)-co-substitution. *ACS Appl. Mater. Interfaces* **2022**, *14*, 20972-20980.
(5) Jia, N.; Cao, J.; Tan, X. Y.; Dong, J.; Liu, H.; Tan, C. K. I.; Xu, J.; Yan, Q.; Loh, X. J.; Suwardi, A. Thermoelectric materials and transport physics. *Mater. Today Phys.* **2021**, *21*, 100519.
(6) Kim, H.-S.; Gibbs, Z. M.; Tang, Y.; Wang, H.; Snyder, G. J. Characterization of Lorenz number with Seebeck coefficient measurement. *APL Mater.* **2015**, *3*, 041506.
(7) Su, H.; Han, Y.; Xie, L.; Jiang, M.; Wang, Z.; Miao, Z.; Liu, G.; Zhou, M.; Huang, R.; Li, L. Fast Fabrication of SnTe via Non-Equilibrium Method and Enhanced Thermoelectric Propertied by Medium-Entropy Engineering. *J. Mater. Chem. C* **2023**.
(8) Nie, C.; Wang, C.; Xu, Y.; Liu, Y.; Niu, X.; Li, S.; Gong, Y.; Hou, Y.; Zhang, X.; Zhang, D.; Li, D.; Zhang, Y.; Tang, G. Band Modification and Localized Lattice Engineering Leads to High Thermoelectric Performance in Ge and Bi Codoped SnTe–$AgBiTe_2$ Alloys. *Small* **2023**, 2301298.
(9) Xu, H.; Wan, H.; Xu, R.; Hu, Z.; Liang, X.; Li, Z.; Song, J. Enhancing the thermoelectric performance of SnTe-$CuSbSe_2$ with an ultra-low lattice thermal conductivity. *J. Mater. Chem. A* **2023**, *11*, 4310–4318.
(10) Xin, X.-Y.; Ma, J.; Liu, H.-Q.; Gu, Y.-J.; Wang, Y.-F.; Cui, H.-Z. A simple Pb-doping to achieve bonding evolution, V_{Sn} and resonant level shifting for regulating thermoelectric transport behavior of SnTe. *J. Mater. Sci. Technol.* **2023**, *151*, 66-72.
(11) Wang, X.; Wu, G.; Wang, R.; Xu, L.; Hu, H.; Sun, P.; Tan, X.; Liu, G.; Jiang, J. Synergistic optimizing thermoelectric performance of SnTe by the integrated Multi-strategy. *Chem. Eng. J.* **2023**, *453*, 139916.
(12) Peng, P.; Wang, C.; Chen, J.; Fan, P.; Du, R.; Si, H.; Cheng, Z.; Wang, J. Enhanced thermoelectric properties of SnTe through core-shell structures and band engineering. *J. Alloy. Compd.* **2023**, *942*, 169010.
(13) Kihoi, S. K.; Shenoy, S. U.; Kahiu, J. N.; Kim, H.; Bhat, D. K.; Lee, H. S. Pushing the limit of synergy in SnTe-based thermoelectric materials leading to an ultra-low lattice thermal conductivity and enhanced ZT. *Sustain. Energy Fuels* **2023**.
(14) Xu, X.; Cui, J.; Huang, Y.; Xia, J.; Pan, K.; Xie, L.; He, J. Microstructural Manipulation for Enhanced Average Thermoelectric Performance: A Case Study of Tin Telluride. *ACS Appl. Mater. Interfaces* **2023**, *15*, 9656-9664.
(15) Zhang, T.; Pan, W.; Ning, S.; Qi, N.; Chen, Z.; Su, X.; Tang, X. Vacancy Manipulation Induced Optimal Carrier Concentration, Band Convergence and Low Lattice Thermal Conductivity in Nano-Crystalline SnTe Yielding Superior Thermoelectric Performance. *Adv. Funct. Mater.* **2022**, 2213761.
(16) Zhang, Q.; Wang, R.; Song, K.; Tan, X.; Hu, H.; Guo, Z.; Wu, G.; Sun, P.; Liu, G.-Q.; Jiang, J. Raised solubility in SnTe by $GeMnTe_2$ alloying enables converged valence bands, low thermal conductivity, and high thermoelectric performance. *Nano Energy* **2022**, *94*, 106940.

(17) Yang, Q.; Lyu, T.; Nan, B.; Tie, J.; Xu, G. Enabling High Quality Factor and Enhanced Thermoelectric Performance in $BiBr_3$-Doped $Sn_{0.93}Mn_{0.1}Te$ via Band Convergence and Band Sharpening. *ACS Appl. Mater. Interfaces* **2022**, *14*, 32236−32243.

(18) Wang, T.; Dou, K.; Wang, H.; Kim, J.; Wang, X.; Su, W.; Chen, T.; Kim, W.; Wang, C. Higher-order anharmonicity leads to ultra-low thermal conductivity and high output power density of SnTe-based thermoelectric materials and modules. *Mater. Today Phys.* **2022**, 100748.

(19) Peng, P.; Wang, C.; Li, L.; Li, S.; Chen, J.; Fan, P.; Du, R.; Si, H.; Cheng, Z.; Wang, J. Enhanced thermoelectric performance of In-doped and AgCuTe-alloyed SnTe through band engineering and endotaxial nanostructures. *Phys. Chem. Chem. Phys.* **2022**, *24*, 27105-27113.

(20) Liu, Y.; Zhang, X.; Nan, P.; Zou, B.; Zhang, Q.; Hou, Y.; Li, S.; Gong, Y.; Liu, Q.; Ge, B.; Cojocaru-Mirédin, O.; Yu, Y.; Zhang, Y.; Chen, G.; Wuttig, M.; Tang, G. Improved Solubility in Metavalently Bonded Solid Leads to Band Alignment, Ultralow Thermal Conductivity, and High Thermoelectric Performance in SnTe. *Adv. Funct. Mater.* **2022**, *32*, 2209980.

(21) Li, M.; Ying, P.; Du, Z.; Liu, X.; Li, X.; Fang, T.; Cui, J. Improved Thermoelectric Performance of P-type SnTe through Synergistic Engineering of Electronic and Phonon Transports. *ACS Appl. Mater. Interfaces* **2022**, *14*, 8171−8178.

(22) Kihoi, S. K.; Shenoy, U. S.; Kahiu, J. N.; Kim, H.; Bhat, D. K.; Lee, H. S. Ultralow Lattice Thermal Conductivity and Enhanced Mechanical Properties of Cu and Sb Co-Doped SnTe Thermoelectric Material with a Complex Microstructure Evolution. *ACS Sustainable Chem. Eng.* **2022**, *10*, 1367−1372.

(23) Abbas, A.; Nisar, M.; Zheng, Z. H.; Li, F.; Jabar, B.; Liang, G.; Fan, P.; Chen, Y.-X. Achieving High Thermoelectric Performance of Eco-Friendly SnTe-Based Materials by Selective Alloying and Defect Modulation. *ACS Appl. Mater. Interfaces* **2022**, *14*, 25802-25811.

(24) Zhang, Q.; Tan, X.; Guo, Z.; Wang, H.; Xiong, C.; Man, N.; Shi, F.; Hu, H.; Liu, G.-Q.; Jiang, J. Improvement of thermoelectric properties of SnTe by MnBi codoping. *Chem. Eng. J.* **2021**, *421*, 127795.

(25) Tian, B.-Z.; Chen, J.; Jiang, X.-P.; Tang, J.; Zhou, D.-L.; Sun, Q.; Yang, L.; Chen, Z.-G. Enhanced Thermoelectric Performance of SnTe-Based Materials via Interface Engineering. *ACS Appl. Mater. Interfaces* **2021**, *13*, 50057-50064.

(26) Pathak, R.; Sarkar, D.; Biswas, K. Enhanced Band Convergence and Ultra-Low Thermal Conductivity Lead to High Thermoelectric Performance in SnTe. *Angew.Chem. Int. Ed.* **2021**, *60*, 17686-17692.

(27) Kihoi, S. K.; Kahiu, J. N.; Kim, H.; Shenoy, U. S.; Bhat, D. K.; Yi, S.; Lee, H. S. Optimized Mn and Bi co-doping in SnTe based thermoelectric material: A case of band engineering and density of states tuning. *J. Mater. Sci. Technol.* **2021**, *85*, 76-86.

(28) Hwang, J.; Lee, M.; Yu, B.-K.; Han, M.-K.; Kim, W.; Kim, J.; Al Rahal Al Orabi, R.; Wang, H.; Acharya, S.; Kim, J.; Jin, Y.; Park, H.; Kim, S.; Yang, S.-H.; Kim, S.-J. Enhancement of thermoelectric performance in a non-toxic $CuInTe_2$/SnTe coated grain nanocomposite. *J. Mater. Chem. A* **2021**, *9*, 14851-14858.

(29) Hong, T.; Wang, D.; Qin, B.; Zhang, X.; Chen, Y.; Gao, X.; Zhao, L.-D. Band convergence and nanostructure modulations lead to high thermoelectric performance in $SnPb_{0.04}Te$-y% $AgSbTe_2$. *Mater. Today Phys.* **2021**, *21*, 100505.

(30) Xu, W.; Yang, H.; Liu, C.; Zhang, Z.; Chen, C.; Ye, Z.; Lu, Z.; Wang, X.; Gao, J.; Chen,

J.; Xie, Z.; Miao, L. Optimized Electronic Bands and Ultralow Lattice Thermal Conductivity in Ag and Y Codoped SnTe. *ACS Appl. Mater. Interfaces* **2021**, *13*, 32876-32885.

(31) Shenoy, U. S.; Bhat, D. K. Bi and Zn co-doped SnTe thermoelectrics: interplay of resonance levels and heavy hole band dominance leading to enhanced performance and a record high room temperature ZT. *J. Mater. Chem. C* **2020**, *8*, 2036-2042.

(32) Ahmad, S.; Singh, A.; Bhattacharya, S.; Navaneethan, M.; Basu, R.; Bhatt, R.; Sarkar, P.; Meshram, K. N.; Debnath, A. K.; Muthe, K. P.; Aswal, D. K. Band Convergence and Phonon Scattering Mediated Improved Thermoelectric Performance of SnTe–PbTe Nanocomposites. *ACS Appl. Energy Mater.* **2020**, *3*, 8882-8891.

(33) Bhat, D. K.; Shenoy, U. S. SnTe thermoelectrics: Dual step approach for enhanced performance. *J. Alloy. Compd.* **2020**, *834*, 155181.

(34) Li, X.; Liu, J.; Li, S.; Zhang, J.; Li, D.; Xu, R.; Zhang, Q.; Zhang, X.; Xu, B.; Zhang, Y.; Xu, F.; Tang, G. Synergistic band convergence and endotaxial nanostructuring: Achieving ultralow lattice thermal conductivity and high figure of merit in eco-friendly SnTe. *Nano Energy* **2020**, *67*, 104261.

(35) Moshwan, R.; Liu, W.-D.; Shi, X.-L.; Sun, Q.; Gao, H.; Wang, Y.-P.; Zou, J.; Chen, Z.-G. Outstanding thermoelectric properties of solvothermal-synthesized $Sn_{1-3x}In_xAg_{2x}Te$ microcrystals through defect engineering and band tuning. *J. Mater. Chem. A* **2020**, *8*, 3978-3987.

(36) Wang, L.; Hong, M.; Sun, Q.; Wang, Y.; Yue, L.; Zheng, S.; Zou, J.; Chen, Z.-G. Hierarchical Structuring to Break the Amorphous Limit of Lattice Thermal Conductivity in High-Performance SnTe-Based Thermoelectrics. *ACS Appl. Mater. Interfaces* **2020**, *12*, 36370-36379.

(37) Zhang, Q.; Guo, Z.; Tan, X.; Mao, L.; Yin, Y.; Xiao, Y.; Hu, H.; Tan, C.; Wu, Q.; Liu, G.-Q.; Xu, J.; Jiang, J. Effects of $AgBiSe_2$ on thermoelectric properties of SnTe. *Chem. Eng. J.* **2020**, *390*, 124585.

(38) Ibáñez, M.; Hasler, R.; Genç, A.; Liu, Y.; Kuster, B.; Schuster, M.; Dobrozhan, O.; Cadavid, D.; Arbiol, J.; Cabot, A.; Kovalenko, M. V. Ligand-Mediated Band Engineering in Bottom-Up Assembled SnTe Nanocomposites for Thermoelectric Energy Conversion. *J. Am. Chem. Soc.* **2019**, *141*, 8025-8029.

(39) Li, D.; Ming, H. W.; Li, J. M.; Zhang, J.; Qin, X. Y.; Xu, W. High Thermoelectric Performance of SnTe via In Doping and $Cu_{1.75}Se$ Nanostructuring Approach. *ACS Appl. Energy Mater.* **2019**, *2*, 8966-8973.

(40) Moshwan, R.; Liu, W.-D.; Shi, X.-L.; Wang, Y.-P.; Zou, J.; Chen, Z.-G. Realizing high thermoelectric properties of SnTe via synergistic band engineering and structure engineering. *Nano Energy* **2019**, *65*, 104056.

(41) Moshwan, R.; Shi, X.-L.; Liu, W.-D.; Wang, Y.; Xu, S.; Zou, J.; Chen, Z.-G. Enhancing Thermoelectric Properties of InTe Nanoprecipitate-Embedded $Sn_{1-x}In_xTe$ Microcrystals through Anharmonicity and Strain Engineering. *ACS Appl. Energy Mater.* **2019**, *2*, 2965-2971.

(42) Bhat, D. K.; Shenoy, U. S. Enhanced thermoelectric performance of bulk tin telluride: Synergistic effect of calcium and indium co-doping. *Mater. Today Phys.* **2018**, *4*, 12-18.

(43) Ma, Z.; Wang, C.; Lei, J.; Zhang, D.; Chen, Y.; Wang, J.; Cheng, Z.; Wang, Y. High Thermoelectric Performance of SnTe by the Synergistic Effect of Alloy Nanoparticles with Elemental Elements. *ACS Appl. Mater. Interfaces* **2019**, *2*, 7354-7363.

(44) Moshwan, R.; Shi, X.-L.; Liu, W.-D.; Yang, L.; Wang, Y.; Hong, M.; Auchterlonie, G.; Zou, J.; Chen, Z.-G. High Thermoelectric Performance in Sintered Octahedron-Shaped $Sn(CdIn)_xTe_{1+2x}$ Microcrystals. *ACS Appl. Mater. Interfaces* **2018**, *10*, 38944-38952.

(45) Bhat, D. K.; Shenoy U, S. High Thermoelectric Performance of Co-Doped Tin Telluride Due to Synergistic Effect of Magnesium and Indium. *J. Phys. Chem. C* **2017**, *121*, 7123-7130.

(46) Al Rahal Al Orabi, R.; Hwang, J.; Lin, C.-C.; Gautier, R.; Fontaine, B.; Kim, W.; Rhyee, J.-S.; Wee, D.; Fornari, M. Ultralow Lattice Thermal Conductivity and Enhanced Thermoelectric Performance in SnTe:Ga Materials. *Chem. Mater.* **2017**, *29*, 612-620.

(47) Al Rahal Al Orabi, R.; Mecholsky, N. A.; Hwang, J.; Kim, W.; Rhyee, J.-S.; Wee, D.; Fornari, M. Band Degeneracy, Low Thermal Conductivity, and High Thermoelectric Figure of Merit in SnTe–CaTe Alloys. *Chem. Mater.* **2016**, *28*, 376-384.

(48) Banik, A.; Shenoy, U. S.; Saha, S.; Waghmare, U. V.; Biswas, K. High Power Factor and Enhanced Thermoelectric Performance of SnTe-$AgInTe_2$: Synergistic Effect of Resonance Level and Valence Band Convergence. *J. Am. Chem. Soc.* **2016**, *138*, 13068-13075.

(49) Tan, G.; Zeier, W. G.; Shi, F.; Wang, P.; Snyder, G. J.; Dravid, V. P.; Kanatzidis, M. G. High Thermoelectric Performance SnTe–In_2Te_3 Solid Solutions Enabled by Resonant Levels and Strong Vacancy Phonon Scattering. *Chem. Mater.* **2015**, *27*, 7801-7811.

(50) Wu, H.; Chang, C.; Feng, D.; Xiao, Y.; Zhang, X.; Pei, Y.; Zheng, L.; Wu, D.; Gong, S.; Chen, Y.; He, J.; Kanatzidis, M. G.; Zhao, L.-D. Synergistically optimized electrical and thermal transport properties of SnTe via alloying high-solubility MnTe. *Energy Environ. Sci.* **2015**, *8*, 3298-3312.

(51) Banik, A.; Shenoy, U. S.; Anand, S.; Waghmare, U. V.; Biswas, K. Mg Alloying in SnTe Facilitates Valence Band Convergence and Optimizes Thermoelectric Properties. *Chem. Mater.* **2015**, *27*, 581-587.

(52) Tan, G.; Shi, F.; Sun, H.; Zhao, L.-D.; Uher, C.; Dravid, V. P.; Kanatzidis, M. G. SnTe–$AgBiTe_2$ as an efficient thermoelectric material with low thermal conductivity. *J. Mater. Chem. A* **2014**, *2*, 20849-20854.
